# Isolate-anchored comparisons reveal evolutionary and functional differentiation across SAR86 marine bacteria

**DOI:** 10.1093/ismejo/wrae227

**Published:** 2024-11-09

**Authors:** Oscar Ramfelt, Kelle C Freel, Sarah J Tucker, Olivia D Nigro, Michael S Rappé

**Affiliations:** Hawai‘i Institute of Marine Biology, School of Ocean and Earth Science and Technology, University of Hawai‘i at Mānoa, Kāne‘ohe, Hawai‘i 96744, United States; Department of Oceanography, School of Ocean and Earth Science and Technology, University of Hawai‘i at Mānoa, Honolulu, HI 96822, United States; Hawai‘i Institute of Marine Biology, School of Ocean and Earth Science and Technology, University of Hawai‘i at Mānoa, Kāne‘ohe, Hawai‘i 96744, United States; Hawai‘i Institute of Marine Biology, School of Ocean and Earth Science and Technology, University of Hawai‘i at Mānoa, Kāne‘ohe, Hawai‘i 96744, United States; Marine Biology Graduate Program, University of Hawai‘i at Mānoa, Honolulu, HI 96822, United States; Department of Marine Science, Hawai`i Pacific University, Waimānalo, HI 96795, United States; Hawai‘i Institute of Marine Biology, School of Ocean and Earth Science and Technology, University of Hawai‘i at Mānoa, Kāne‘ohe, Hawai‘i 96744, United States

**Keywords:** SAR86, Magnimaribacter, marine bacteria, phylogenomics, proteorhodopsin

## Abstract

SAR86 is one of the most abundant groups of bacteria in the global surface ocean. However, since its discovery over 30 years ago, it has remained recalcitrant to isolation and many details regarding this group are still unknown. Here, we report the cellular characteristics from the first SAR86 isolate brought into culture, *Magnimaribacter mokuoloeensis* strain HIMB1674, and use its closed genome in concert with over 700 environmental genomes to assess the phylogenomic and functional characteristics of this order-level lineage of marine *Gammaproteobacteria*. The SAR86 order *Magnimaribacterales* invests significant genomic resources into the capacity for $\beta$-oxidation, which is present in most genomes with high gene copy numbers. This cyclical set of reactions appears to be fed by components of cell membranes that include lipids such as phosphatidylcholine, phosphatidylethanolamine, glycolipids, and sulfolipids. In addition to the widespread capacity to degrade the side chain of steroidal compounds via $\beta$-oxidation, several SAR86 sublineages also appear able to fully degrade the steroid polycyclic ring structure as well as other aromatic, polycyclic, and heterocyclic molecules. Read recruitment from publicly available metagenomes reveals that the *Magnimaribacterales* compose up to 6% of the global surface ocean microbial community. Only a subset of genera drives these high relative abundances, with some more globally dominant and others restricted to specific oceanic regions. This study provides an unprecedented foundation through which to understand this highly abundant yet poorly understood lineage of marine bacteria and charts a path to bring more representatives of this order into laboratory culture.

## Introduction

The marine bacterial lineage known as SAR86 was initially discovered over 30 years ago through the application of 16S rRNA gene sequencing to identify microorganisms within natural communities of marine plankton^1^; “SAR” for its discovery in a surface seawater sample from the Sargasso Sea, and “86” for clone 86. Soon after its discovery within the open-ocean gyres of the Atlantic and Pacific Oceans,^1-3^ diverse relatives of the original gene clone were recovered from around the global ocean, including coastal systems.^4-9^ Reports on the abundance of SAR86 by counting cells via fluorescence in situ hybridization, quantitative polymerase chain reaction (PCR), and other molecular approaches routinely support the view that it is one of the most abundant bacteria in seawater, particularly in the surface ocean.^10-16^

In a landmark study, one of the first applications of environmental genomics revealed a gene encoding the photoactive transmembrane protein proteorhodopsin in a SAR86 genome fragment.^17^ While research on proteorhodopsin has flourished,^18-21^ a comprehensive picture of SAR86 marine bacteria has been slower to develop. In this study, we provide the first characterization of SAR86 cells in culture and leverage the closed genome sequence of the isolated SAR86 strain in a set of analyses designed to elucidate the evolutionary and functional characteristics of SAR86 bacteria. Despite the ubiquity of SAR86 cells in the global ocean, there is currently little to link extensive 16S rRNA gene-based studies from the past three decades with the genomic diversity uncovered over the past two decades,^12^^,^^22-25^ and thus no existing backbone from which to interpret relationships between phylogenetic diversity, environmental distribution, and the distribution of functional traits encoded by genomes. Thus, included in our goals were to carefully investigate the HIMB1674 genome along with hundreds of publicly available SAR86 environmental genomes in order to evaluate the evolutionary origins of SAR86 within the *Gammaproteobacteria*, as well as evolutionary relationships between the strain genome and other SAR86 environmental genomes. We also sought to identify the genomic features that typify HIMB1674 and other SAR86 bacteria, as well as features that may distinguish major sublineages within SAR86. Finally, by recruiting sequence reads from publicly available metagenomes from marine plankton, we aimed to provide the first comprehensive analysis of unifying evolutionary history with the environmental distribution of SAR86 genotypes.

Through this study, we propose the name *Magnimaribacter mokuoloeensis* gen. nov., sp. nov., for the first SAR86 cultivar. The genus name, *Magnimaribacter* is derived from the Latin words “magni” (here, great in quantity) and “mare” (sea). The species name, *Mokuoloeensis*, refers to the island (Moku o Loʻe) on which the laboratory is located where the strain was isolated. Accordingly, we also propose a new family, *Magnimaribacteraceae* fam. nov., to encompass the SAR156 lineage that contains *M. mokuoloeensis* and a new order, *Magnimaribacterales* ord. nov., to encompass all of the SAR86 lineage.

## Materials and methods

### Strain isolation, genome sequencing, and electron microscopy

The methods used to isolate strain HIMB1674 were initially described elsewhere.^26^ Seawater intended for growth medium was collected on 8 July 2017 from Kāneʻohe Bay (N 21° 27.699, W 157° 47.010) and was prepared following previously published methods.^27^ Briefly, a 20 L seawater sample was collected in acid-washed 4 L polycarbonate (PC) bottles from a depth of 2 m at station SR4 (N 21° 27.699′, W 157° 47.010′). Within 1 h of collection, the seawater was sequentially filtered through prerinsed (10 L of sterile water followed by 10 L of seawater) 0.8, 0.2, and 0.1 μm-pore-sized polyethersulfone (PES) membranes (AcroPak 20 and Supor 100; Pall Corp., Port Washington, NY, USA) into clean 4 L PC bottles. Bottles were then autoclaved for 3 h at 121°C and allowed to cool. The sterile seawater was sparged with CO_2_ to restore the inorganic carbon chemistry, and then with air, through three in-line HEPA vent filters (0.3 μm glass fiber to 0.2 μm polytetrafluoroethylene (PTFE) to 0.1 μm PTFE; Whatman, GE Healthcare Life Sciences, Chicago, IL, USA) and stored at 4°C until use. The pH of the seawater was checked prior to being autoclaved and after being sparged to ensure continuity of the inorganic carbon chemistry. A 4 L raw seawater sample was then collected in an acid-washed PC bottle on 26 July 2017 from station SB (N 21° 26.181′, W 157° 46.64196′) within Kāneʻohe Bay, Oʻahu, Hawaiʻi, and used as inoculum for the high throughput cultivation experiment. The raw seawater was diluted in the previously prepared sterile seawater medium to yield a cellular concentration of 2.5 cells ml^−1^, aliquoted in 2 ml volumes into six 96-well custom-made deep well Teflon plates, and incubated at 27°C in the dark. Between 3 and 8 weeks after inoculation, the plates were screened for growth with a guava easyCyte 5HT flow cytometer equipped with a high-powered 150 mW blue (488-nm) laser (Millipore, Burlington, MA, USA) following a previously published protocol.^28^ All wells with growth of >10^4^ cells ml^−1^ were sub-cultured into 20 ml of fresh seawater medium, amended with 400 mM (NH4)2SO4, 400 mM NH4Cl, 50 mM NaH2PO4, 1 mM glycine, 1 mM methionine, 50 mM pyruvate, 800 nM niacin (B3), 425 nM pantothenic acid (B5), 500 nM pyridoxine (B6), 4 nM biotin (B7), 4 nM folic acid (B9), 6 mM myo-inositol, 60 nM 4-aminobenzoic acid, and 6 mM thiamine hydrochloride (B1). Cultures were then re-incubated and subsequently screened again. Sub-samples of growing cultures were cryopreserved with a 10% final concentration of glycerol, as well as filtered for DNA extraction, through a 0.03 μm PES membrane (18 ml of HIMB1674 at a cellular concentration of 6.76 × 105 cells ml^-1^). Extracted genomic DNA was used to identify cultures via PCR amplification and subsequent MiSeq (Illumina, San Diego, California) based sequencing of a fragment of the 16S rRNA gene using barcoded 515Y and 926R primers that target the V4 and V5 regions of the 16S rRNA gene.^26^^,^^29^ To more precisely determine the identity of strain HIMB1674, the full length 16S rRNA gene was also amplified, sequenced, and a phylogenetic tree created as described in the Supplementary Information.

The genome of strain HIMB1674 was sequenced (paired-end, 2x151 bp) on a NextSeq 2000 (Illumina, San Diego, California, USA) from a library constructed using a modified NextEra XT library protocol^30^ and 7 ng of genomic DNA that was extracted from the original 18 ml of culture described above. After quality control using Trimmomatic v.0.36,^31^ reads were assembled using metaSPAdes v.3.13.0.^32^

Scanning and transmission electron photomicrographs were prepared as described in the Supplementary Information.

### Environmental genomic data

Publicly available environmental genomes affiliated with the SAR86 lineage were identified within the “Genome Taxonomy Database” (GTDB)^33^ and PATRIC database,^34^ and subsequently downloaded from GenBank. Within the GTDB, all genomes found under the bacterial order tag “o__SAR86” were downloaded on 22 June 2020. The PATRIC database was searched for potential SAR86 genomes using the search term “SAR86” on 27 July 2020. Lastly, genomes characterized as SAR86 by the study of Zhou *et al.*^23^ were downloaded from GenBank. All genomes downloaded from GenBank utilized the bit program v1.8.17.^35^

Genomes were initially checked for quality and level of completion using the CheckM v1.1.2 taxonomy workflow and parameters for the class *Gammaproteobacteria.*^36^ Of 280 marker genes, 252 were found within the closed HIMB1674 genome (Table 1, Table S1). The 28 marker genes missing from HIMB1674 were subsequently excluded from a second iteration of the CheckM workflow to generate completion values based on HIMB1674, the only closed SAR86 genome standard. Genomes that were >80% complete and contained <5% contamination based on this modified core marker gene set were retained for subsequent downstream analyses. The presence of the 280 *Gammaproteobacteria* marker genes was subsequently assessed for all genomes of the SAR86 lineage (Table S1).

**Table 1:** Summary of the genomic characteristics of *M. mokuoloeensis* str. HIMB1674 and the four families of the order *Magnimaribacterales*.

	HIMB1674	Suzuki	CHAB-I-7	RedeBAC7D11	*Magnimaribacteraceae*
No. genomes (species set/ expanded species set)^a^	1/1	39/93	7/10	19/59	10/25
No. genera	na	10	4	4	4
No. genes present in the 280-gene marker set	252	240	228	241	251
% completion, conserved 252-gene set (ave. ±SD)	100	87.4 ± 4.4	84.2 ± 1.9	86.2 ± 4.0	87.1 ± 5.5
±SD)c	100	91.9 ± 4.7	94.6 ± 2.2	90.9 ± 4.0	87.4 ± 5.4
Est. genome size, 252-gene set (ave. ±SD; Mbp)	1.62	1.39 ± 0.13	1.18 ± 0.07	1.30 ± 0.06	1.66 ± 0.10
Est. genome size, family-specific gene sets (ave. ±SD; Mbp)	1.62	1.32 ± 0.14	1.05 ± 0.06	1.24 ± 0.06	1.65 ± 0.10
No. genes, actual (ave. ±SD)	1643	1267 ± 166	1029 ± 50	1165 ± 65	1505 ± 153
GC content (ave. ±SD; %)	36.4	34.6 ± 2.9	34.4 ± 1.1	34.4 ± 0.7	36.3 ± 0.5

a Number of genomes in the SAR86 species dataset consisting of representatives from >93% ANI species clusters/number of genomes in the expanded species dataset consisting of genomes <99% ANI to their representative species cluster genome

A Kruskal-Wallis statistical analysis was used to compare genome size across major SAR86 phylogenetic lineages and was run using the R (v4.1.2)^37^ method “Kruskal test” from its built-in stats package. A follow-up Dunn’s post-hoc analysis was performed using rstatix v0.7.2^38^ on the data when statistically significant differences (*P* < .05) were found in order to identify where these differences were located. This methodology was also used to compare the GC% content between different sublineages.

A recent publication reported on the assembly of complete SAR86 genomes from collections of closely related SAGs.^22^ We attempted to verify the veracity of these assemblies and discovered them to be artificial chimeras resulting from extensive misassembly. We describe this further in the Supplementary Information (Supplementary text and Table S8). Thus, while some of the findings of this previous study are consistent with our current observations, we maintained reliance on the individual high-quality SAGs as they appear in their original publications so as to avoid artifacts arising from artificial, chimeric genomes.

### Phylogenomic analyses

Genomes were initially grouped into species clusters following a previously published approach^39^ using an “average nucleotide identity” (ANI) value of 93% as described in the Supplementary Information, with representative genomes from each species cluster referred to as the SAR86 species dataset (*n* = 75 genomes, Table S2). A Proteobacteria-wide phylogeny utilizing the SAR86 species dataset and reference genomes (Table S3) was created in order to verify that these genomes form a monophyletic grouping to the exclusion of other *Gammaproteobacteria* and to assess the evolutionary origins of the SAR86 lineage within the *Gammaproteobacteria*. Additional details are provided in the Supplementary Information.

To identify comparable taxonomic levels across sublineages of the SAR86 clade, the SAR86 species dataset was used, excluding genomes identified as not belonging to the SAR86 lineage. A “relative evolutionary distance” (RED) approach was then used to identify taxonomic levels within the SAR86 lineage.^40^ Additional details are provided in the Supplementary Information.

In order to investigate evolutionary relationships within the SAR86 clade, an additional phylogenomic analysis using a broadened SAR86 dataset that included genomes sharing <99% ANI with their assigned species cluster representative, referred to as the SAR86 expanded dataset (*n* = 185 genomes; Table S2), was performed. The SAR86 expanded dataset and outgroup genomes were processed using the GTDB-Tk “identify” and “align” commands in the same manner as the *Gammaproteobacteria*-wide phylogeny. No reference genomes from GTDB were included during the alignment step. The alignment file was then used to infer a tree using ModelFinder within IQ-Tree,^41^ which determined the best-fit model to be LG + F + R7 and included 1000 ultrafast bootstrap replicates. Genomes from members of the *Gammaproteobacteria* orders *Burkholderiales* and *Pseudomonadales* were used as an outgroup. Additional details of the phylogenomic analyses are provided in the Supplementary Information.

### Comparative genomics and read recruitment

The anvi’o pangenome workflow v7.1^42^^,^^43^ was used with the SAR86 expanded genome dataset to identify patterns in gene content. Metagenomic read recruitment was used to investigate the abundance of SAR86 genomes across two publicly available open-ocean sampling endeavors: TARA Oceans^44^ and GEOTRACES^45^ (Table S4). The approach of Shaiber and colleagues^46^ was used to estimate the abundance of SAR86 genomes. Additional details are provided in the Supplementary Information.

## Results

### Strain HIMB1674 are small cells that possess small genomes typical of oligotrophic marine bacteria

**Figure 1:**
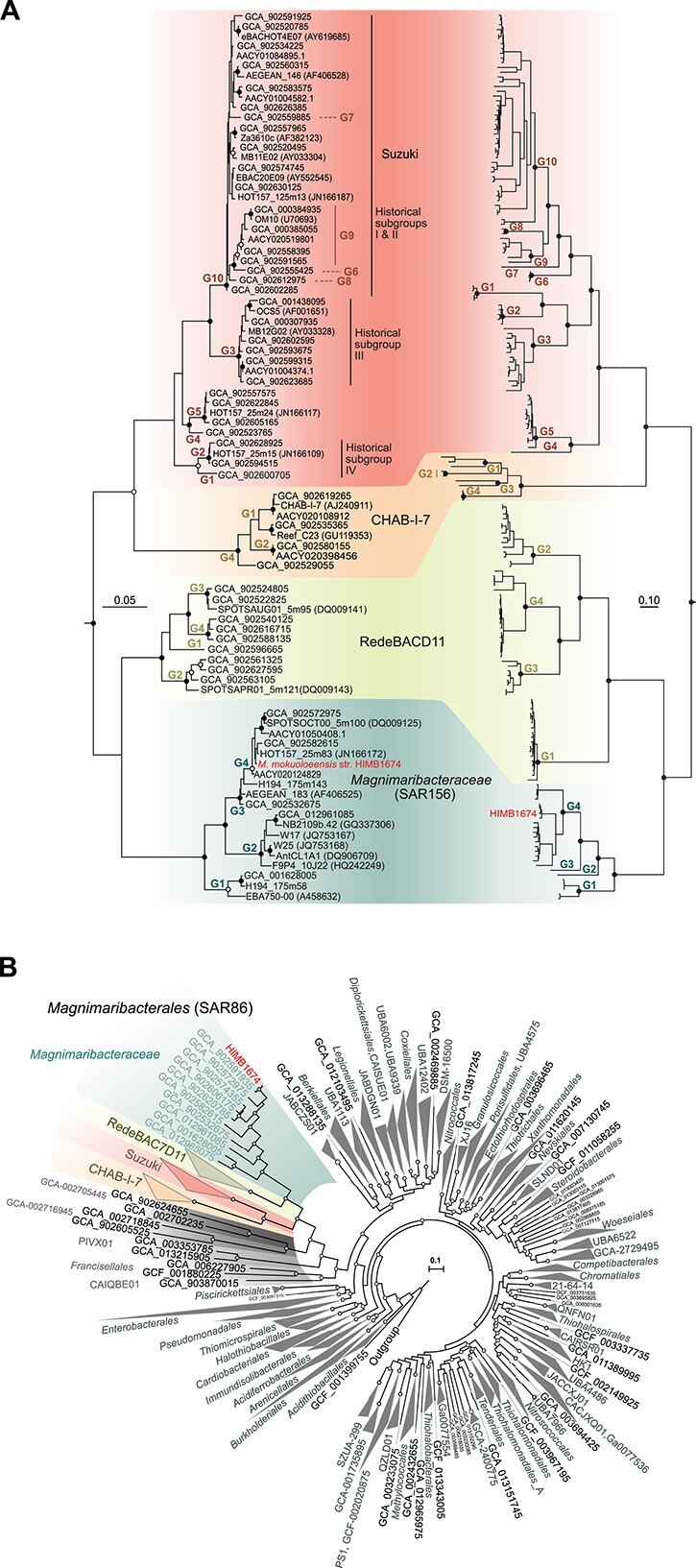
Phylogenetic affiliations of strain HIMB1674. (A) Comparison between a 16S rRNA gene phylogeny (left) and a phylogenomic analysis using a concatenated alignment of 120 single-copy core genes (right). *Magnimaribacter mokuoloeensis* str. HIMB1674 diverges at the root of SAR86 within the SAR156 subclade, now the family *Magnimaribacteraceae*. Historical subgroups CHAB-I-7 and RedeBAC7D1 are shown, along with historical subgroups I-IV that together make up the family-level Suzuki lineage. “G” denotes genus-level groupings defined by phylogenomics and RED values. Solid circles indicate bootstrap values greater than 95%, whereas open circles indicate values over 80%. The 16S rRNA gene phylogeny was constructed from rRNA genes extracted from the SAR86 expanded genome dataset with historical sequences included for reference. *Betaproteobacteria* 16S rRNA gene sequences were used as an outgroup. The phylogenomic analysis included members of the gammaproteobacterial orders *Burkholderiales* and *Pseudomonadales* as an outgroup (GCF_000305785.2, GCF_003752585.1, GCF_003574215.1, GCF_006980785.1). For phylogenomic analysis, support value circles were removed from below genus nodes to maintain clarity on branching patterns. (B) Phylogenetic placement of the SAR86 order *Magnimaribacterales* within the *Gammaproteobacteria*. Genomes from 416 family-level lineages of Proteobacteria from GTDB release 202 and 75 genomes from the *Magnimaribacterales* order were included. The phylogeny is rooted in the internal node distinguishing the *Gammaproteobacteria* from the other three classes *Alphaproteobacteria*, *Magnetococcia*, and *Zetaproteobacteria*. Circles indicate ultrafast bootstrap support values ≥95% from 1000 replicates.

**Figure 2:**
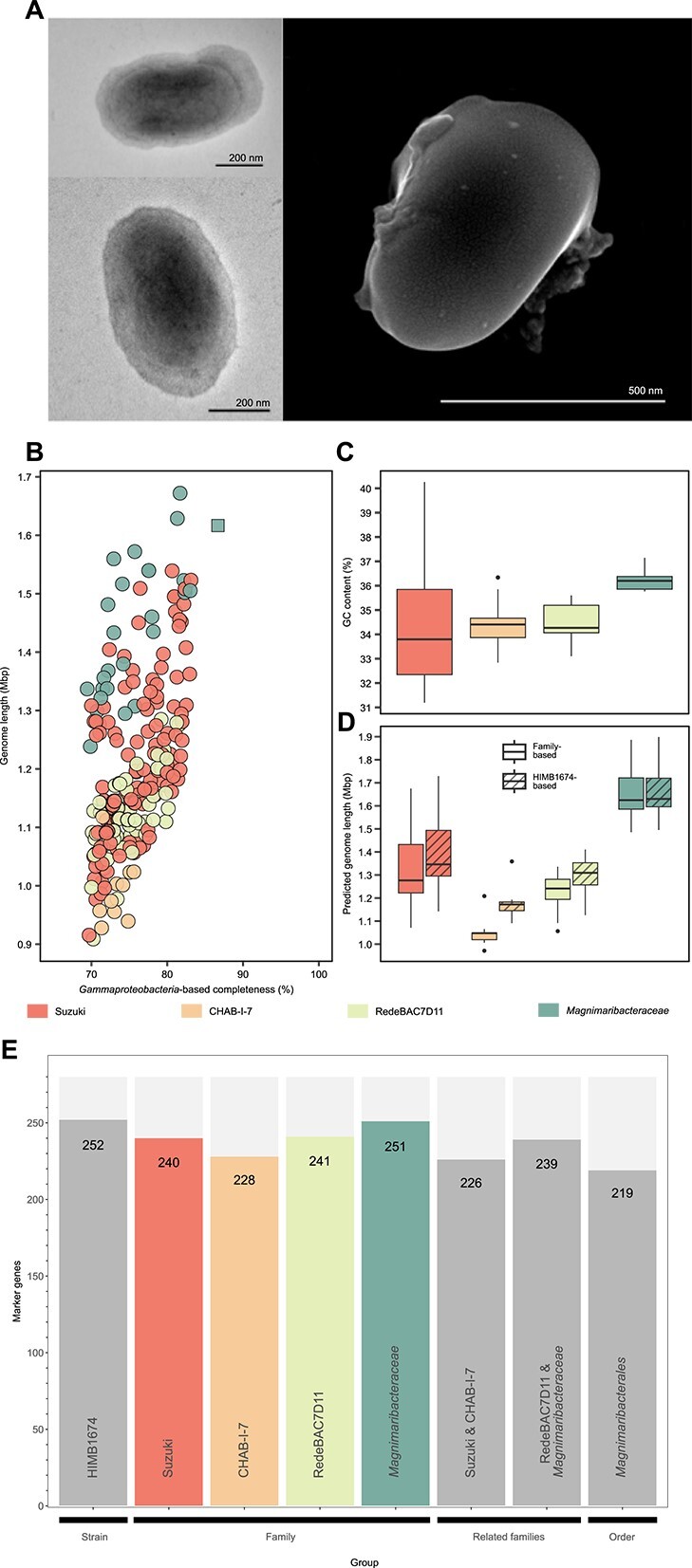
Characteristics of *M. mokuoloeensis* str. HIMB1674 and high-quality SAR86 environmental genomes. *(A)* TEM and SEM images reveal uniform small coccobacilli in a growing monoculture of strain HIMB1674. (B) Distribution of genome completeness based on the GTDB *Gammaproteobacteria* marker gene set compared with absolute genome length. HIMB1674 is indicated by a square. (C) The different families within the SAR86 order *Magnimaribacterales* harbor distinct %GC values. (D) The different families within the *Magnimaribacterales* also harbor distinct predicted genome lengths, calculated with the *Gammaproteobacteria* marker genes present in the complete HIMB1674 isolate genome as well as marker genes present within the high-quality genomes of each family. (E) Barchart showing the number of marker genes from the *Gammaproteobacteria* dataset present for different groupings. The SAR86 expanded genome dataset (*n* = 185) used in panels B–E includes 25 *Magnimaribacteraceae*, 57 RedeBAC7D11, 10 CHAB-I-7, and 93 Suzuki genomes.

A large dilution-to-extinction cultivation experiment using seawater from the coastal Hawaiʻi surface ocean resulted in 339 isolates that were then identified via short-read 16S rRNA gene sequencing. The affiliation of one strain (HIMB1674) with the SAR86 clade was confirmed by full-length 16S rRNA gene sequencing (Fig. 1A). Whole genome sequencing yielded a single closed scaffold of 1.62 Mbp in length and a GC content of 36.4% containing 1699 total genes (1643 protein-coding), one rRNA operon, and a coding density of 96.3% (Table 1) Scanning and transmission electron micrographs consistently revealed strain HIMB1674 to be uniformly small spheroid bacilli (0.46 ± 0.03 × 0.66 ± 0.11 μm; estimated biovolume of 0.07 ± 0.01 μm^3^) (Fig. 2A). We name this strain *M. mokuoloeensis*.

### A reduced marker gene set differentiates SAR86 from other *Gammaproteobacteria*

Although the genome of *M. mokuoloeensis* str. HIMB1674 is closed; it lacks 28 of 280 genes in the marker gene set used for *Gammaproteobacteria* in CheckM’s taxonomic-specific workflow^36^ (Table 1), yielding a completion value of only 86.7%. The missing marker genes are affiliated with a spectrum of functions, including several related to DNA recombination and repair (Table S1). Nearly all (27 of 28) of the missing genes are also systematically absent from a dataset of 732 putative environmental SAR86 genomes (Table S1). A conservative correction of the 28 marker genes missing from the HIMB1674 genome led to an increase in the average completion of the initial dataset of 705 putative environmental SAR86 genomes from just under 60% to 68% (Table S2).

### SAR86 is a diverse lineage that branches deeply within the *Gammaproteobacteria*

From the initial dataset of 732 genomes, 224 were retained for downstream analyses following quality control (>80% complete and <5% contamination) using the modified marker gene set of 252 genes (“Passed Quality Check” in Table S2). Together with the closed *M. mokuoloeensis* str. HIMB1674 genome, this was further reduced to a set of 75 genomes that represent >93% ANI genome clusters (SAR86_species_rep in Table S2), which we functionally refer to as species clusters.

A Proteobacteria-wide phylogenomic analysis constructed with a site-specific frequency model that included 771 reference genomes and the 75 genomes of the SAR86 species dataset revealed that nearly all of the putative SAR86 genomes grouped within a single monophyletic clade that includes HIMB1674 (Fig. 1B), with the exception of three genomes originating from the SAR86 “marine subsurface clade” described by Zhou et al.^23^ that group within the bacterial order *Pseudomonadales*, where they are closely related to the marine gammaproteobacterial lineage OM182^8^ (data not shown). The SAR86 lineage is a deep, order-level branch within the *Gammaproteobacteria*, where it shares a common evolutionary origin with two lineages composed exclusively of metagenome-assembled genomes (MAGs) generated from marine metagenomes, labeled GCA-002705445 and PIVX01 in GTDB release 202 (Fig. 1B). These three lineages of marine origin together share a common ancestry with the GTDB order CAIQBE01 that consists of two MAGs from freshwater lakes, and the order Francisellales, which includes a variety of cultured and uncultured microorganisms including representatives from freshwater and marine systems. We have named the SAR86 branch of *Gammaproteobacteria* the bacterial order *Magnimaribacterales*.

Using the SAR86 species dataset again (but without the *Pseudomonadales*-related genomes), taxonomic ranks assigned from RED values yielded four families and 22 genera within the SAR86 order *Magnimaribacterales* (Fig. S1), which is broadly consistent with the four families and 18 genera recognized in the GTDB. Cross-referencing with the 16S rRNA gene-based phylogeny revealed that three of the four family-level lineages corresponded to previously defined SAR86 subclades including RedeBAC7D11,^47^^,^^48^ which has also been referred to as SPOTS^15^ (AG-339-G14 in GTDB), CHAB-I-7^47^^,^^49^ (TMED112 in GTDB) and SAR156^2^^,^^50^ (SAR86 in GTDB), which contains *M. mokuoloeensis* (Fig. 1). The fourth family-level lineage (D2472 in GTDB) consisted of the previously identified SAR86 subclades I, II, III, and IV.^47^^,^^51^ In order to provide a meaningful label for this family-level lineage we use “Suzuki” after Marcelino T. Suzuki, in recognition of his influential research characterizing the diverse lineages of the SAR86 clade.^47^^,^^51^ We also retain the original labels of RedeBAC7D11 and CHAB-I-7 for these families for consistency with previously published literature and name the SAR156 family-level lineage the *Magnimaribacteraceae* after the type species *M. mokuoloeensis* str. HIMB1674.

Phylogenomic analyses revealed that the family-level lineages Suzuki and CHAB-I-7 share a common evolutionary origin, as do the *Magnimaribacteraceae* and RedeBAC7D11 families (Fig. 1). While the SAR86 clade appears to possess a rapid evolutionary clock relative to most other *Gammaproteobacteria*, this is particularly true for the CHAB-I-7 family (Fig. 1). Based on the geographic origin of genomes, the four families are widely distributed across the global ocean, and are mostly limited vertically to the ocean surface layer (Table S2). The *Magnimaribacteraceae* family is an exception; genomes from two genera of *Magnimaribacteraceae* (Ma-G1 and Ma-G2) originate exclusively from below the photic zone; in contrast, the other two (Ma-G3 and HIMB1674-containing *Magnimaribacter*) are affiliated with the surface ocean (Table S2). This unique aspect of the *Magnimaribacteraceae* is corroborated by 16S rRNA gene analysis that revealed several lineages that originate from below the photic zone and in the deep ocean (Fig. S2).

### Small genomes and low %GC typify SAR86 bacteria

An assessment of genome completeness based on the GTDB *Gammaproteobacteria* conserved gene set revealed completion values of all environmental SAR86 genomes, barring those that belonged to the *Pseudomonadales* order, to be less than the 86.7% of HIMB1674 (Fig. 2B). For this reason, we calculated modified completion estimates based off of the marker gene set adapted from the HIMB1674 genome and verified across 732 environmental SAR86 genomes. Using the marker gene set conserved in HIMB1674, genomes within the four families of the *Magnimaribacterales* order averaged 1.18–1.66 Mbp (Fig. 2D, Table 1, Table S2). However, an analysis of the marker gene set individually across the four families within the *Magnimaribacterales* revealed that the likely number of missing marker genes ranged from 29 to 52 and was conserved at the family level (Table 1, Table S1). When applying marker gene sets specific to each family, the average genome size decreases to 1.05 Mbp for CHAB-I-7 (Fig. 2D). The GC content of SAR86 genomes range from 31% to 40% (Table S2), with some families and genera possessing distinct %GC (Fig. 2C, Fig. S3).

A Kruskal-Wallis statistical test with a Dunn’s post-hoc test found that predicted genome sizes, corrected based on the conserved gene marker set, were significantly different between all four families within the SAR86 order *Magnimaribacterales* (*P* < .05). Genome sizes estimated from the family-specific marker gene sets were also found to be significantly different (*P* < .05). Using the same statistical approach, we found that the %GC was significantly different between the *Magnimaribacteraceae* and other three families (*P* < .05), but no statistically significant differences were found between the three remaining families (*P* > .05).

### β-Oxidation is central to the organic carbon metabolism of the SAR86 order *Magnimaribacterales*

**Figure 3:**
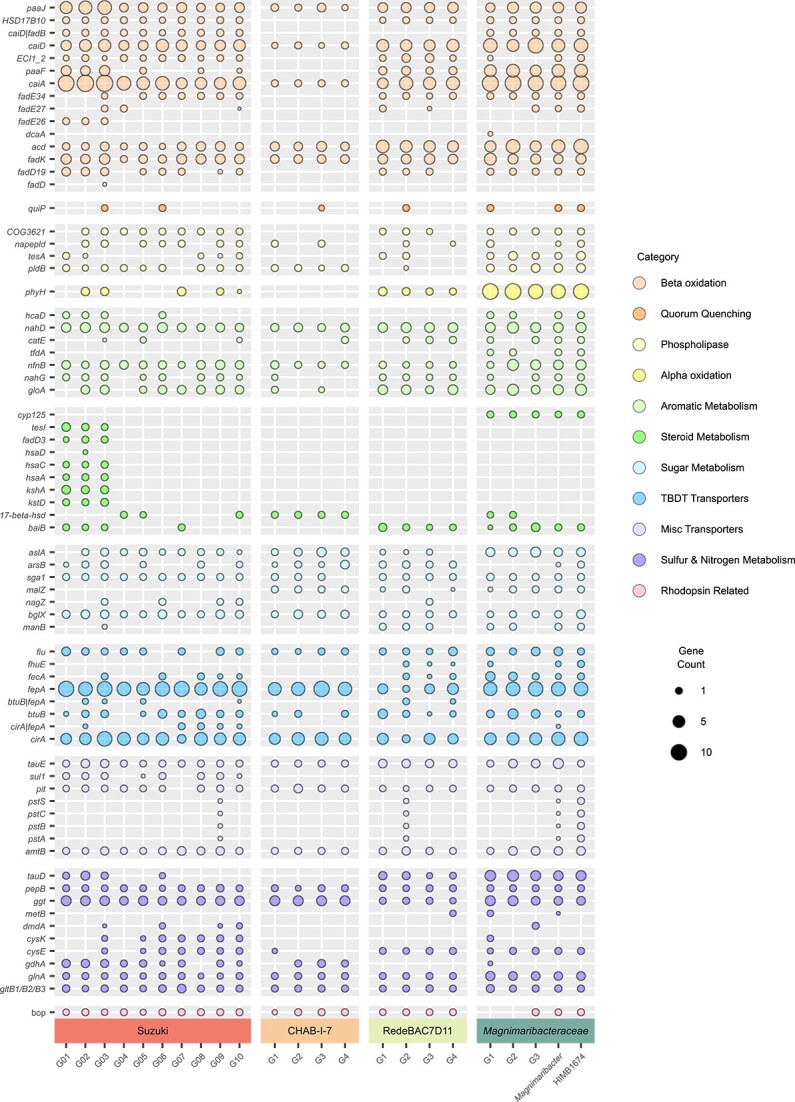
Distribution of key metabolic features across the bacterial order *Magnimaribacterales*. The size of each circle indicates the average per genome abundance of each gene within a genus. A comprehensive table of this data is provided in Table S5.

Consistent with the genome of strain HIMB1674, all four families within the SAR86 order *Magnimaribacterales* harbor the components necessary for a functional TCA cycle, glyoxylate shunt, and complete 2-methylcitrate cycle (Fig. 3, Table S5), except genomes of the CHAB-I-7 family where no gene encoding a methylisocitrate lyase (*prpB*) was identified. *M. mokuoloeensis* and other members of the SAR86 order *Magnimaribacterales* encode a complete “Embden-Meyerhof-Parnas” (EMP) glycolytic pathway, except that no gene encoding glucose-6-phosphate isomerase (*pgi*) was identified in genomes of roughly half of the RedeBAC7D11 and *Magnimaribacteraceae* families (Fig. 3, Table S5).

Genes encoding enzymes that carry out the initiation and four steps of the $\beta$-oxidation pathway for fatty acid degradation are present in some of the highest copy numbers within the *M. mokuoloeensis* genome, and are also widespread across the entire SAR86 order *Magnimaribacterales* (Fig. 3, Table S5). However, no identifiable 3-hydroxyacyl-CoA dehydrogenase gene required to fulfill step 3 (*fadB*) is found within any of the genomes of the CHAB-I-7 family, which also do not contain elevated copy numbers for this pathway. *M. mokuoloeensis* also encodes the ability to degrade the fatty acid-like side chains of steroidal compounds, including genes with homology to 3-oxocholest-4-en-26-oate-CoA hydrogenase (*fadE26*,*27*) and 3-oxochol-4-en-24-oyl-CoA hydrogenase (*fadE34*), that are also broadly present across the families *Magnimaribacteraceae*, Suzuki, and RedeBAC7D11 (Fig. 3, Table S5). Similarly, *M. mokuoloeensis* contains putative Δ3,Δ2-enoyl-CoA isomerases (*ECI1_2*) that are also present in a majority of *Magnimaribacteraceae*, Suzuki, and RedeBAC7D11 genomes suggesting that *M. mokuoloeensis* and these three families may share the ability to metabolize unsaturated fatty acids. This expands the variety of substrates that these lineages may degrade via $\beta$-oxidation.

The entire SAR86 order *Magnimaribacterales* appears able to obtain fatty acids from lipids through putative phospholipases encoded in the genomes of all four families. A subset of genomes also encode putative quorum quenching genes, such as those for acyl-homoserine lactone acylases (*quiP*) that have the potential to provide another source of fatty acids (Fig. 3). Finally, genes encoding putative phytanoyl-CoA dioxygenases (*phyH*) were identified within *M. mokuoloeensis* as well as other *Magnimaribacteraceae*, Suzuki, and RedeBAC7D11 family genomes, revealing the potential ability to degrade the phytol sidechain of chlorophyll or similar compounds via $\alpha$-oxidation (Fig. 3, Table S5).

### Aromatic and steroidal core ring cleavage is widespread across the SAR86 order *Magnimaribacterales*

In addition to its fatty acid side chain, a diverse set of genes encoding enzymes involved in the catabolism of the core rings of steroid-like compounds were identified across the *Magnimaribacterales* order (Fig. 3, Table S5). These include genes within all CHAB-I-7 and a portion of the Suzuki family that encode putative 3 (or 17) beta hydroxysteroid dehydrogenases (*17-beta-hsd*) known to be involved in the degradation of sterols. In contrast, a majority of genomes from the RedeBAC7D11 and *Magnimaribacteraceae* families contain genes bearing homology to bile acid-coenzyme A ligases (*baiB*) that are known to play an important role in the degradation of bile acids,^52^ which also possess a steroidal ring structure. Genes bearing homology to cholest-4-en-3-one 26-monooxygenases (*cyp125*), which participate in cholesterol ring degradation, were also identified in a majority of genomes within the *Magnimaribacteraceae* family. Several genomes within the Suzuki family contain genes encoding almost all the enzymes required to degrade androstenedione-like compounds into 3-[(3aS,4S,7aS)-7a-methyl-1,5-dioxo-octahydro-1H-inden-4-yl] propanoate and 2-hydroxy-cis-hex-2,4-dienoate. 2-hydroxy-cis-hex-2,4-dienoate can be further degraded through the catechol meta-cleavage pathway (described below). Genes for this particular pathway (*tesl*, *hsaA,C,D*, *fadD3*, *kshA*, *kstD*) were not detected in other genomes of the Suzuki family, or in the genomes of other families of *Magnimaribacterales* (Fig. 3, Table S5).

In addition to steroidal compounds, the capacity to degrade aromatic compounds is likely widespread across the SAR86 order *Magnimaribacterales* (Fig. 3, Table S5), and genomic evidence indicates that simple polycyclic aromatic hydrocarbons may be used as substrates. Genes that encode putative 2-hydroxychromene-2-carboxylate isomerases (*nahD*), which are involved in the degradation of naphthalene to salicylate, are widespread across the *Magnimaribacterales*. In addition, genes encoding putative salicylate hydroxylases (*nahG*) that convert salicylate into catechol are present in a majority of genera from the Suzuki, RedeBAC7D11, and *Magnimaribacteraceae* families. Genes encoding catechol 2,3 dioxygenases (*catE*/*gloA*), crucial to the meta-cleavage pathway of catechol degradation in order to complete aromatic ring cleavage, are a near-universal feature of SAR86 genomes and usually present in multiple copies. In addition to the widespread genes described above, the *Magnimaribacteraceae* family possesses genes encoding enzymes involved in aromatic compound degradation that were less widely distributed across other members of the order. This includes genes related to putative 3-phenylpropionate/trans-cinnamate dioxygenases (*hcaD*) involved in the metabolism of trans-cinnamate and genes related to 2,4-dichlorophenoxyacetate dioxygenases (*tfdA*) that are linked to the degradation of 2,4-dichlorophenoxyacetic acid.^53^ The potential for bacteria of the *Magnimaribacterales* order to metabolize nitroaromatic substrates is supported by the presence of genes encoding putative nitroreductases (*nfnB*) that may degrade a broad range of nitroaromatic compounds, resulting in the release of nitrogen compounds such as ammonium (NH_4_) and potentially leading to the further degradation of the remaining aromatic end product via the gene complements described above.^54^

Sugar metabolism enzymes involved in the degradation of oligo- and polysaccharides are also encoded by *Magnimaribacterales* order genomes (Fig. 3, Table S5). Although a majority of *Magnimaribacterales* encode glucoamylases (*sga1*) and beta-glucosidases (*bglX*), other enzymes are restricted in distribution. For example, genes encoding putative endo-1,4-beta-mannosidases (*manB*) are found across a majority of RedeBAC7D11 and *Magnimaribacteraceae* genomes but are largely absent from the other two *Magnimaribacterales* families. Genes encoding putative alpha-glucosidases (*malZ*) are present in the CHAB-I-7, RedeBAC7D11, and *Magnimaribacteraceae* families but missing from the Suzuki family. Conversely, the Suzuki family is the only family-level lineage that contains genes encoding putative beta-N-acetylhexosaminidases (*nagZ*) across multiple genera. Although the genomes of a single genus (Re-G3) within the RedeBAC7D11 family also contain genes encoding putative beta-N-acetylhexosaminidases, they are not present within genomes of the CHAB-I-7 or *Magnimaribacteraceae* families. All families within the SAR86 order *Magnimaribacterales* also contain putative arylsulfatases (*aslA*/*arsB*) that are potentially involved in the desulfation of sulfoglycolipids.^55^

### TonB-dependent transporters dominate the *Magnimaribacterales* transporter pool

Genes encoding TonB-dependent transporters (TBDTs) were abundant across all families of the SAR86 order *Magnimaribacterales* (Fig. 3, Table S5). Of these, two were particularly abundant: *cirA*, an iron-catecholate receptor, and *fepA*, a transporter for ferrienterochelin, the iron complex of the siderophore enterobactin. *FepA* has also been shown to transport enterobactin^56^ and some colicins.^57^ Other TBDTs that are widespread across SAR86 bacteria include *fiu*, a receptor for the iron siderophore ferrichrome A and monomeric catechols, and *fecA*, a receptor for ferric citrate that was particularly prevalent across the RedeBAC7D11 and *Magnimaribacteraceae* families. The TBDT for vitamin B12, *btuB*, is also found throughout the *Magnimaribacterales* order.

Genes encoding putative ammonia (*amtB*) and inorganic phosphate (*pit*) transporters are a core feature of genomes from across the *Magnimaribacterales* order. Less widespread is the PsT system of high-affinity phosphate transporters that are present within a subset of genomes from the family RedeBAC7D11 as well as the *M. mokuoloeensis* genome. Although several transporters with annotations related to sugar transport are widespread across SAR86 genomes, they also contain annotations suggesting an alternative role in the transport of cyclic compounds, including, shikimate and lipids such as sphingosine-1-phosphate (Fig. 3, Table S5).

### Ammonia and organic sulfur are likely sources of cellular nitrogen and sulfur across the *Magnimaribacterales*

Consistent with the presence of the ammonia channel protein (*amtB*) gene, mechanisms for nitrogen assimilation via ammonia are present across genomes of the *Magnimaribacterales* order. One pathway includes putative glutamate synthase (*gltB1*/*B2*/*B3*) and glutamine synthetase (*glnA*) genes, which support the GS-GOGAT pathway (Fig. 3). The second mechanism, via putative glutamate dehydrogenases (*gdhA*), is almost exclusive to the Suzuki and CHAB-I-7 families (Fig. 3, Table S5).

Although no support for sulfate incorporation is present within SAR86 genomes outside of a putative sulfate permease across the Suzuki family, various alternative potential sources to fulfill cellular sulfur requirements were encoded by SAR86 genomes (Fig. 3, Table S5). All four families within the *Magnimaribacterales* order possessed putative gamma-glutamyl transpeptidases (*ggt*) and leucyl aminopeptidases (*pepB*) for converting glutathione into cysteine. In addition, almost all RedeBAC7D11 and *Magnimaribacteraceae* family genomes, as well as a subset of Suzuki, possess putative taurine dioxygenases (*tauD*) that may convert taurine into sulfite and other byproducts. However, all *Magnimaribacterales* order genomes analyzed here lack a recognizable sulfite reductase (*dsrA*, *cysJ*, *asrA*, *sir*) needed to convert sulfite into sulfide, and cysteine synthase (*cysK*) is largely limited to genomes from Suzuki and *Magnimaribacteraceae* genus Ma-G1. This suggests that the *Magnimaribacterales* order is either unable to utilize the sulfite produced from taurine, utilize an unrecognized pathway for its utilization, or the putative taurine dioxygenase serves a different function. Although cystathionine gamma-synthases (*metB*) would provide such an alternative method to incorporate sulfite, putative homologs are only present in genomes from RedeBAC7D11 and *Magnimaribacteraceae*, and not present across the Suzuki and CHAB-I-7 families. Finally, genes encoding putative dimethylsulfoniopropionate demethylases (*dmdA*) may confer the ability to degrade dimethylsulfoniopropionate (DMSP) within a subset of genera primarily from the Suzuki family, providing another sulfur source for this limited number of genomes.

### *Magnimaribacterales* bacteria are primarily associated with surface seawater and makeup ~2–6% of the global ocean microbial community

**Figure 4:**
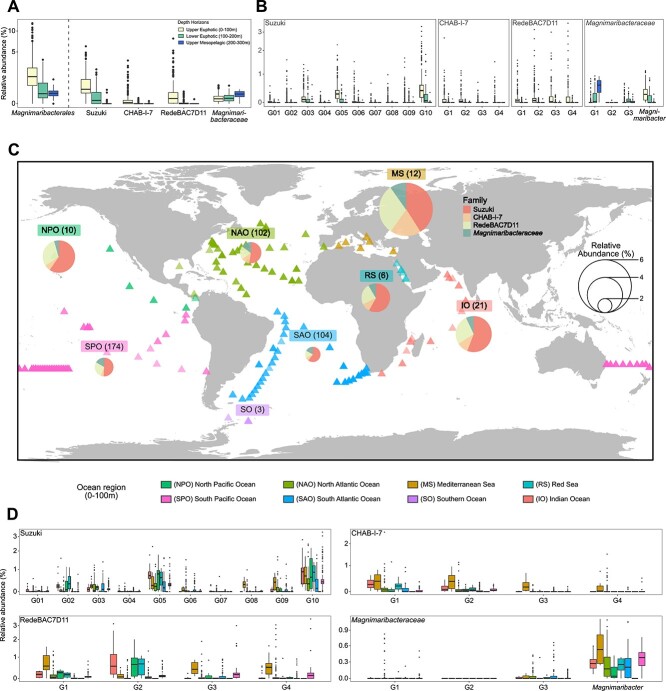
Relative abundance of the bacterial order *Magnimaribacterales* across the global ocean. (A) Percent of reads recruited from TARA (*n* = 144) and GEOTRACES (*n* = 462) metagenomes originating from the uppermost 300 m of the water column were mapped to genomes from across the SAR86 species dataset, revealing maximum abundance within the surface layer (<100 m). This was mirrored by the abundance of three of four families, with only the *Magnimaribacteraceae* increasing in abundance with depth. (B) Genus-level view of the read recruitment shown in panel a, highlighting differences in the distribution of genera and the prevalence of Ma-G1 with depth. Relative abundance of *Magnimaribacterales* families (C) and genera (D) within the upper 100 m of the water column across the global ocean, using the read recruitment data shown in panel A. The numbers above each pie chart indicate the number of metagenomes utilized for the average relative abundance calculation from each region, whereas the size of the pie chart indicates the relative abundance of *Magnimaribacterales* in the particular oceanic region. Slices correspond to the relative abundance of each of the four families. Colored triangles indicate TARA oceans and GEOTRACEs metagenome sample locations, with fill color corresponding to their associated ocean region.

Across a global survey of metagenomes spanning the upper 300 m of the water column, read recruitment reveals that the relative abundance of the SAR86 order *Magnimaribacterales* is higher on average in the surface ocean above 100 m and decreases with depth (Fig. 4A) for most families. However, the *Magnimaribacteraceae* family is unique in that the relative abundance of the family increases with depth (Fig. 4A). The depth-specific distribution is uneven across the four genera of the *Magnimaribacteraceae* family, with two genera peaking below the upper (0–100 m) euphotic zone (Fig. 4B). Across all four families, relative abundance is unevenly distributed across genera, particularly within the Suzuki family, where some genera are either consistently more abundant across the global ocean, or abundant only in particular regions (Fig. 4D). For example, several genera that are largely absent from all other oceanic regions peak in abundance within metagenomes from the Mediterranean Sea.

Within the 0–100 m depth horizon, the relative abundance of the four families within the *Magnimaribacterales* order varies between different regions of the global ocean (Fig. 4C, Table S6). Although the Mediterranean Sea harbors the greatest average relative abundance of SAR86 (Magnimaribacterales) bacteria (~6%), other heavily sampled ocean regions such as the North Atlantic Ocean, South Atlantic Ocean, and South Pacific Ocean harbor fairly uniform abundances of 2%–3%. SAR86 bacteria are largely absent from the Southern Ocean. The average relative abundances of individual families within the *Magnimaribacterales* order are stable across the global ocean, with the Suzuki family the most abundant across all oceanic regions.

Read recruitment to metagenomes that encompassed different size fractions did not reveal a clear trend that would indicate either extremely small cells, or those potentially associated with larger particles such as marine snow or ecto- or endosymbionts of larger cells (Fig. S4).

## Discussion

Isolating the first strain of the SAR86 marine bacterial clade allowed us to assess the cellular, evolutionary, functional, and genomic characteristics of SAR86 within the context of a closed genomic template for the first time. A phylogenomic approach that accounted for the low %GC content of SAR86 genomes and included hundreds of newly available SAR86 single amplified genomes revealed that SAR86 forms an order-level lineage, the *Magnimaribacterales*, that branches deeply within the *Gammaproteobacteria* in a similar fashion to its SAR11 counterparts within the *Alphaproteobacteria*, the *Pelagibacterales.*^58^ Also consistent with other ubiquitous marine bacterial lineages such as the *Pelagibacterales*, *Magnimaribacterales* 16S rRNA genes form a broad clade composed of several sublineages widely distributed in the global ocean.^51^^,^^59^^,^^60^ However, historical 16S rRNA gene-based analyses have long revealed uncertainty with regard to affiliations within SAR86, and even what sublineages may or may not be part of this order (e.g.^15^^,^^47^^,^^50^). Our phylogenomic analyses clarify the presence of four robust family-level lineages within the SAR86 order *Magnimaribacterales*, including the previously described CHAB-I-7 and RedeBAC7D11 families, the Suzuki family that includes previously described SAR86 subclades I through IV under its umbrella, and the SAR156 sublineage, which we describe here as the family *Magnimaribacteraceae* that contains *M. mokuoloeensis* str. HIMB1674. While historical studies have been inconsistent with their inclusion of the SAR156 sublineage within SAR86 (e.g.^15^^,^^47^^,^^61^; Supplementary Information), our phylogenomic analyses are consistent with the GTDB taxonomy and conclusively demonstrate that it forms a sister subclade to RedeBAC7D11 within the SAR86 order *Magnimaribacterales*.

The capacity for $\beta$-oxidation was previously identified within a small set of incomplete SAR86 genomes.^12^^,^^23^ Here, we show that the $\beta$-oxidation pathway is found in multiple copies across all four families of the order (including within the genome of isolate HIMB1674.) We interpret the dedication of such a significant proportion of otherwise small genomes as an indication of the importance of this process to the core metabolism of SAR86 bacteria. Within individual genomes, the multiple gene copies at several steps of the $\beta$-oxidation pathway harbor considerable sequence variation. We hypothesize that these variants expand the variety of substrates available for degradation by this pathway. The presence of diverse lipases, including, phospholipases, reveals the likely utilization of lipids as an organic substrate, with liberated fatty acids feeding into the $\beta$-oxidation pathway. Further, it is likely that the capacity to import and degrade a variety of lipid head groups or their components is widely distributed across SAR86 bacteria. This includes multiple genes encoding glycosidases that we predict localize within the periplasm and target glycolipids, including lipopolysaccharides, in order to cleave the oligo- or polysaccharide moiety, and genes encoding predicted arylsulfatase A enzymes capable of cleaving sulfate from sulfolipids prior to their further degradation into labile subunits. The sum of these reactions is the periplasmic degradation of complex lipids to generate relatively simple subunits that are subsequently available for import and degradation within the cytoplasm of SAR86 cells.

We were surprised to discover evidence that some SAR86 cells may also have the capacity to catabolize components of steroidal compounds that, like lipids, are an important constituent of cell membranes. The ability to metabolize the side chain of the core four-ring steroid system and feed it through a series of $\beta$-oxidation reactions, similar to the degradation of lipid-derived fatty acids, appears to be a common trait of SAR86 bacteria, with the exception of the CHAB-I-7 family. Perhaps more surprising is the presence of a small number of genera within the Suzuki family that possess the genetic potential to also fully degrade the core steroid rings and, in addition to these few genera, the broad distribution of several genes associated with the conversion or degradation of steroidal rings across the *Magnimaribacterales* order. This suggests that the majority of SAR86 cells may also be able to catabolize at least a portion of the generally recalcitrant steroidal core.

Beyond steroid-like compounds, extensive genomic evidence indicates that cells of the *Magnimaribacterales* order target other aromatic, heterocyclic, or polycyclic compounds for catabolism. The widespread presence of catechol 2,3-dioxygenases indicates that the simple aromatic catechol is likely a significant substrate or intermediate for aromatic compound degradation, while the presence of ring-cleaving nitroreductases indicates that nitroaromatic compounds may also play an important role in *Magnimaribacterales* metabolism. Other lines of genomic evidence support our assertion that SAR86 cells specialize in the degradation of aromatic compounds. For example, the presence of 2-oxoglutarate ferredoxin oxidoreductases in an alternative TCA cycle, as found in the RedeBAC7D11 and *Magnimaribacteraceae* families, has been shown to facilitate the reduction of aromatic rings in other bacteria via an increase in production of reduced ferredoxin.^62^ Glutathione S-transferases are known to play a role in the sequestration and transport of endogenous hydrophobic compounds, which include steroids and other aromatic, heterocyclic, or polycyclic compounds. We hypothesize that the high copy number of genes encoding glutathione S-transferases across the SAR86 order *Magnimaribacterales* is a function of the important role they play in rendering some of these hydrophobic compounds more hydrophilic and thus accessible as substrates for catabolism by *Magnimaribacterales* cells. The high abundance of TonB-dependent transporters predicted to import iron-containing siderophores is also consistent with the high iron demand of enzymes involved in the predicted metabolisms of SAR86 bacteria, as iron is a common requirement of the ring-cleaving mono- and dioxygenases found in abundance across the order. Catechol serves as a subunit for several of the siderophores likely targeted by TonB receptors from the *Magnimaribacterales* order, raising the intriguing possibility that these molecules are targeted for both iron and as a source of organic carbon.

Although SAR86 bacteria are primarily a surface ocean-dwelling lineage of planktonic marine bacteria, the *Magnimaribacteraceae* family contains some sublineages that peak in relative abundance within the aphotic zone. Here, we show that two of four genus-level sublineages within this family are most abundant in the subsurface ocean, while the other two (including the genus *Magnimaribacter*) are most abundant in the surface ocean. In support of this observation, genes encoding the light-driven proton pump proteorhodopsin common to surface ocean-dwelling SAR86 genomes were not identified within genomes of the two genera of “deep” *Magnimaribacteraceae*. This analysis thus provides a framework from which to investigate the depth-specific distribution of *Magnimaribacterales* order genomes and the genetic determinants of ecotypic differentiation with depth.

Across the global ocean, the relative abundance of SAR86 genomes in the upper 200 m of the water column ranged from an average of nearly 6% in the Mediterranean Sea to being absent from the Southern Ocean. However, SAR86 16S rRNA gene sequences have been found in seawater from both the Arctic and Antarctic.^63^ Despite this variation in abundance, the relative proportions of the four families remained fairly stable across all ocean regions, with Suzuki making up around half, RedeBAC7D11 making up around one fourth and CHAB–I-7 and the *Magnimaribacteraceae* making up a little over 10% each of the global SAR86 community. This intriguing observation indicates that, although the proportion of *Magnimaribacterales* within a planktonic community may vary in relative abundance, the response of individual families is relatively stable and thus potentially predictable. Within each of the four *Magnimaribacterales* families, a small subset of genus-level lineages was the primary drivers of the relative abundance within each group. A unique exception to this was a select few genus-level lineages from Suzuki, CHAB-I-7, and RedeBAC7D11 that were almost completely absent from all other regions of the global ocean except for the Mediterranean Sea. The presence of unique Mediterranean lineages is not exclusive to SAR86 bacteria, as it resembles previous observations of *Pelagibacterales* marine bacteria.^64^ These Mediterranean-specific sublineages also do not appear to directly compete with other closely related sublineages, as many of the most abundant sublineages found in the North Pacific and Indian Oceans are found at similar relative abundances in the Mediterranean. These results suggest that particular sublineages of the *Magnimaribacterales* possess characteristics adapted to niches only present in the Mediterranean.

The closed genome of *M. mokuoloeensis* str. HIMB1674 lacked 10% of genes in the *Gammaproteobacteria* conserved marker gene set commonly used by the GTDB to assess a variety of genome quality parameters, including estimated completion. This caused us to critically assess the presence of this marker set across the hundreds of environmental genomes investigated in this study, whereby we discovered that SAR86 genomes were broadly lacking nearly all (27 of 28) of the same makers, with additional marker sets consistently absent from each family. While the standard *Gammaproteobacteria* conserved marker gene set thus underestimates the completeness of SAR86 genomes and overestimates their predicted length, this is particularly true for the CHAB-I-7 lineage. This family systematically lacked 18% of the marker gene set that, when accounted for in completion estimates, results in genome length estimates of 1 Mbp. Intrigued by the unusually small genome size and fast evolutionary clock of this family relative to other SAR86 genomes, we used read recruitment to different size fractions of marine plankton to tentatively conclude that cells of the CHAB-I-7 lineage are free-living bacteria and not unusually small in cell size or affiliated with larger cells. Coupled with apparent lesions in multiple metabolic pathways that are otherwise shared by other members of the *Magnimaribacterales*, these unique genomic characteristics make the CHAB-I-7 family a valuable target for understanding genome streamlining and the limits to genome reduction in presumably free-living microorganisms.

*Magnimaribacterales* and *Pelagibacterales* marine bacteria are two of the most abundant groups of heterotrophs inhabiting the global surface ocean where they share characteristic streamlined, low %GC genomes that harbor significant evolutionary divergence and concomitant genomic variation within their respective deep branching lineages of proteobacteria. Although genome-sequenced isolates have facilitated the dissection of *Pelagibacterales* metabolism for over two decades, our study represents the first report of a SAR86 isolate, its cellular characteristics, and its associated complete genome sequence.

One likely factor contributing to the cohabitation of *Magnimaribacterales* and *Pelagibacterales* cells is that they occupy different niche spaces. For heterotrophic bacteria in seawater, the diversity of compounds within the organic matter pool offers a plethora of opportunities for specialization, and thus, niche differentiation. While SAR11 has come to be known as a specialist in metabolizing labile, low-molecular-weight dissolved organic matter (DOM),^60^ we show that SAR86 potentially targets a distinct and surprising fraction of the DOM, including lipid and steroid components of cell membranes. The *Magnimaribacterales* are also likely capable of metabolizing other aromatic, polycyclic, and heterocyclic substrates. While the identity of these compounds is not yet known, hints gleaned from the gene annotations reveal catechol-based siderophores, chlorophyll, and nucleosides as potential targets. Although they may not be commonly thought of as abundant in oceanic systems, direct evidence reveals that some of these potential organic carbon substrates are present within the exo- or endometabolite pools of marine systems.^65-67^ However, much of our metabolic inference is based on pathways described from human pathogens and microbes within other systems that are often distantly related to planktonic marine bacteria,^68^ leaving open the opportunity describing specific substrates and the mechanisms by which *Magnimaribacterales* cells access and degrade this unique and frequently hydrophobic fraction of the global DOM pool. Although we acknowledge that *M. mokuoloeensis* strain HIMB1674 is not yet sufficiently domesticated to the point where we are able to routinely assay for substrate utilization, the combination of complete genome sequence and an isolated strain provides the necessary tools needed to further this work.

**Protologue (Table S7)**

###  

#### Description of *Magnimaribacter* gen. nov.

*Magnimaribacter* (Magni.mari.bac’ter. N.L. masc. n. magni from the Latin magnitudo, which can mean great in importance or number and mari from the Latin mare or Maris, which means sea). Cells are small, non-motile coccobacilli, chemoorganotrophic, and found as free-living planktonic cells inhabiting seawater.

The type species is *M. mokuoloeensis*.

### Description of *M. mokuoloeensis* sp. nov.

*Magnimaribacter mokuoloeensis* (moku.o.loe.en’sis. N.L. masc. n. Moku o Loʻe island located in Kāneʻohe Bay, Oʻahu, Hawaiʻi, and is where the first isolate of this lineage was cultivated, the name of this island in ʻŌleo Hawaiʻi literally translates to the island of Loʻe).

The type strain, HIMB1674^T^, was isolated from seawater of the tropical Pacific Ocean at 2 m water depth within Kāneʻohe Bay on the island of Oʻahu, Hawaiʻi. The DNA G + C content of the type strain is 36.4 mol% (determined from the genome sequence), and the genome is 1.6 Mb in length. The GenBank accession number for the genome sequence of strain HIMB1674^T^ is CP151794.

### Description of *Magnimaribacteraceae* fam. nov.

*Magnimaribacteraceae* (Magni.mari.bac’ter.a.ce’ae. N.L. masc. n. *Magnimaribacter*, type genus of the family; suff. -aceae, ending to denote a family; N.L. fem. pl. n. *Magnimaribacteraceae*, the family of the genus *Magnimaribacter*).

Description of the family *Magnimaribacteraceae* is the same as for the genus *Magnimaribacter*. The type genus is *Magnimaribacter*. The family includes the SAR156 phylogenetic clade.

### Description of *Magnimaribacterales* ord. nov.

*Magnimaribacterales* (Magni.mari.bac’ter.a.ce’ae. N.L. masc. n. *Magnimaribacter*, type genus of the order; suff. -ales, ending to denote an order; N.L. fem. pl. n. *Magnimaribacterales*, the order of the genus *Magnimaribacter*).

Description of the order *Magnimaribacterales* is the same as for the genus *Magnimaribacter*. The type genus is *Magnimaribacter*. The order encompasses the SAR86 phylogenetic clade of marine bacteria.

## Supplementary Material

ramfelt_supplementary_Figure_1_wrae227

ramfelt_supplementary_Figure_2_wrae227

ramfelt_supplementary_Figure_3_wrae227

ramfelt_supplementary_Figure_4_wrae227

ramfelt_supplementary_Figure_5_wrae227

ramfelt_supplementary_table_1_wrae227

ramfelt_supplementary_table_2_wrae227

ramfelt_supplementary_table_3_wrae227

ramfelt_supplementary_table_4_wrae227

ramfelt_supplementary_table_5_wrae227

ramfelt_supplementary_table_6_wrae227

ramfelt_supplementary_table_7_wrae227

ramfelt_supplementary_table_8_wrae227

ramfelt_supplementary_11042024

## Data Availability

The assembled HIMB1674 genome and raw sequencing reads are available at NCBI BioProject PRJNA1098794. The code used for analysis is available on GitHub (https://github.com/kolaban/isolated-anchor-comparisons-sar86-isme-pub) linked to a Zenodo repository (10.5281/zenodo.13897608).
