# Supplementary figures and images for: Isolate-anchored comparisons reveal evolutionary and functional differentiation across SAR86 marine bacteria
Source: ISME J. 2024 Nov 9;18(1):wrae227. doi: 10.1093/ismejo/wrae227 (PMC11582366; doi:10.1093/ismejo/wrae227)

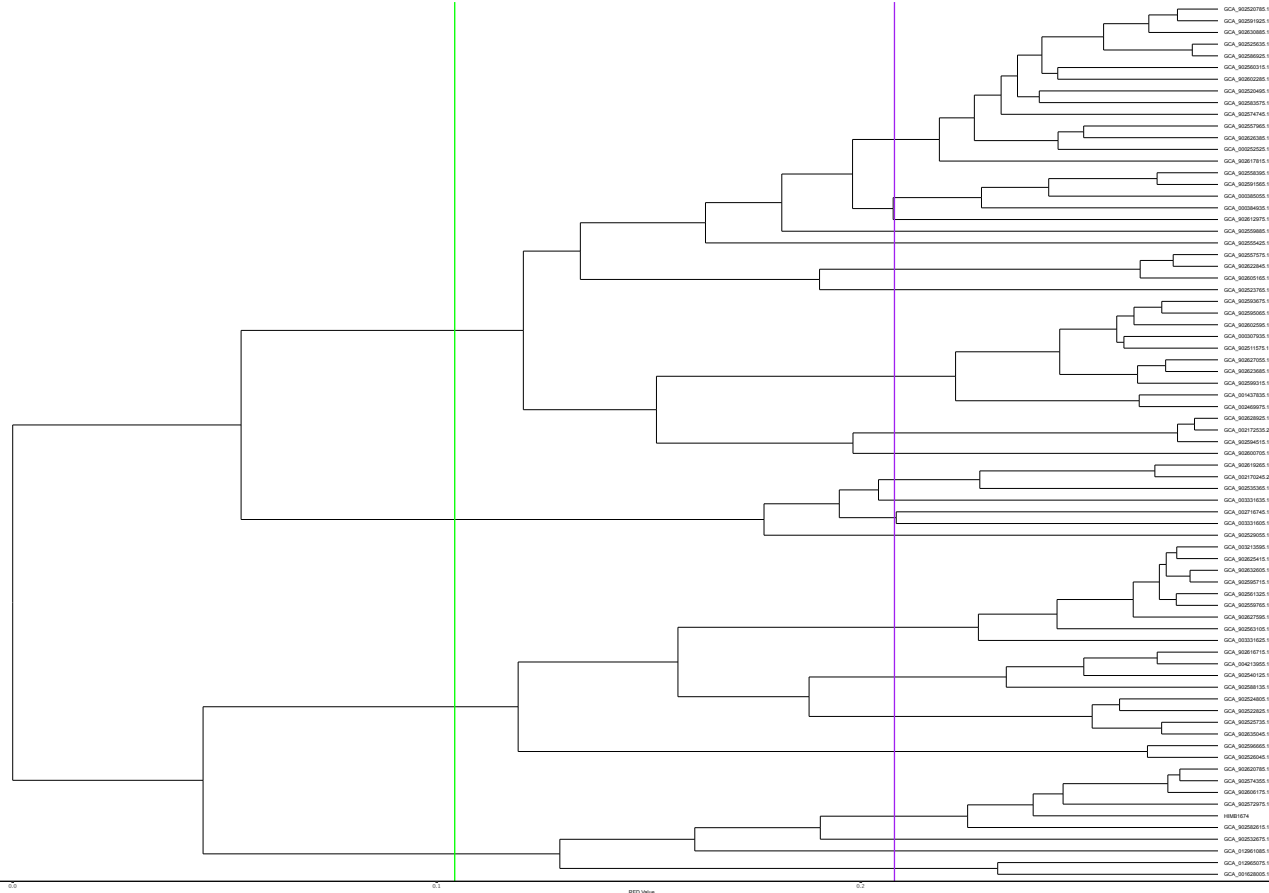

Supplement: ramfelt_supplementary_Figure_1_wrae227 [file ramfelt_supplementary_figure_1_wrae227.pdf]

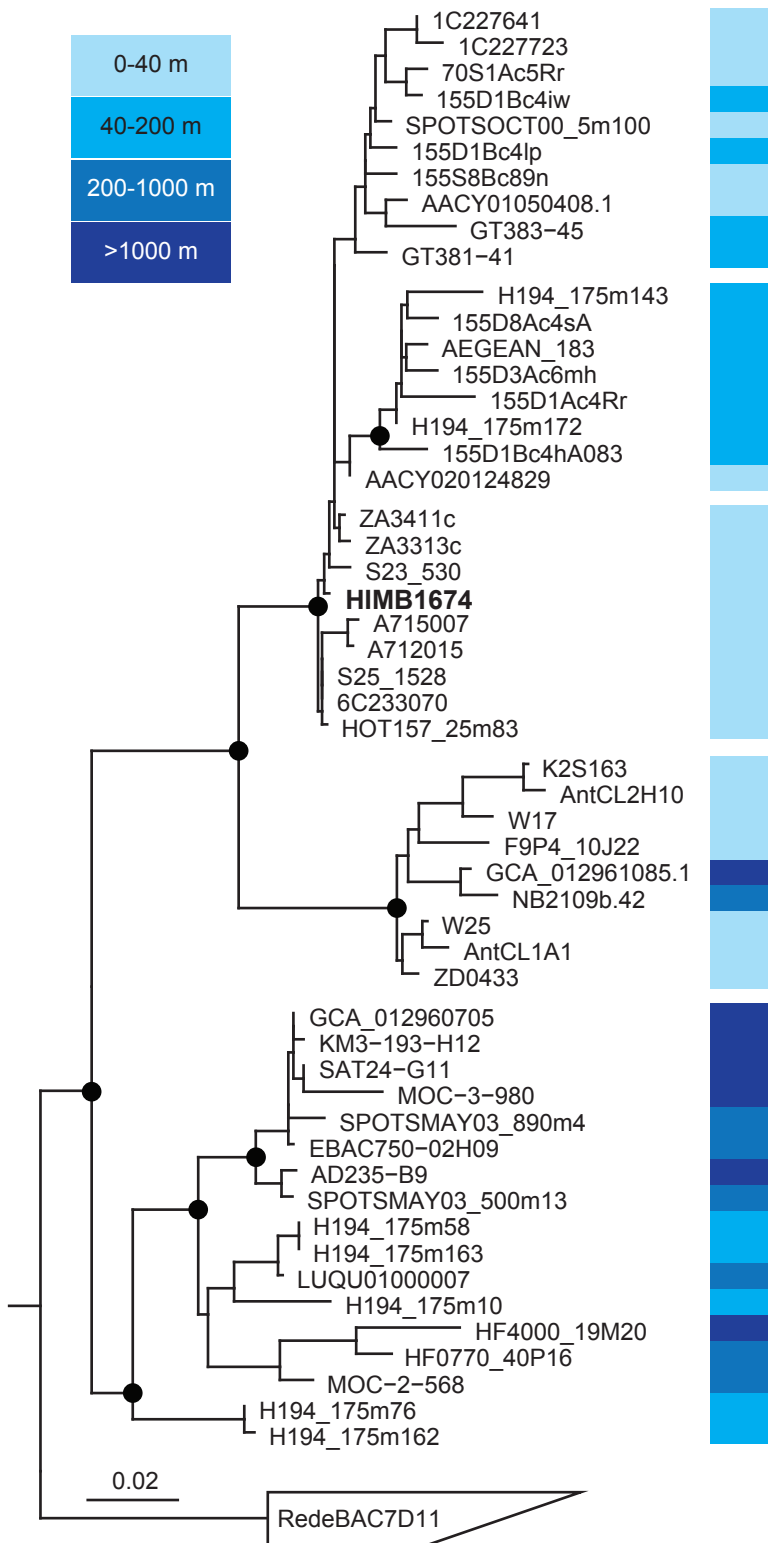

Supplement: ramfelt_supplementary_Figure_2_wrae227 [file ramfelt_supplementary_figure_2_wrae227.pdf]

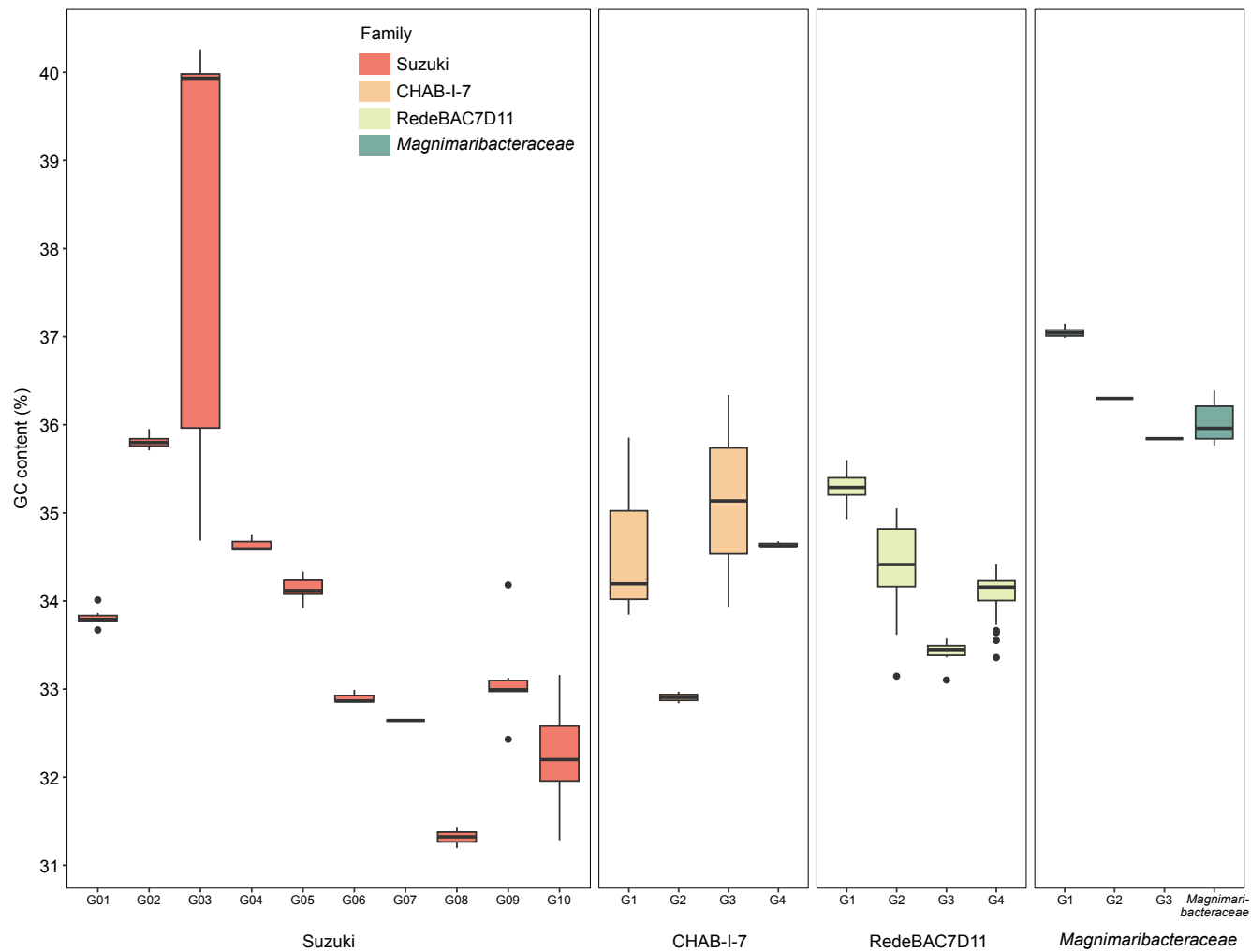

Supplement: ramfelt_supplementary_Figure_3_wrae227 [file ramfelt_supplementary_figure_3_wrae227.pdf]

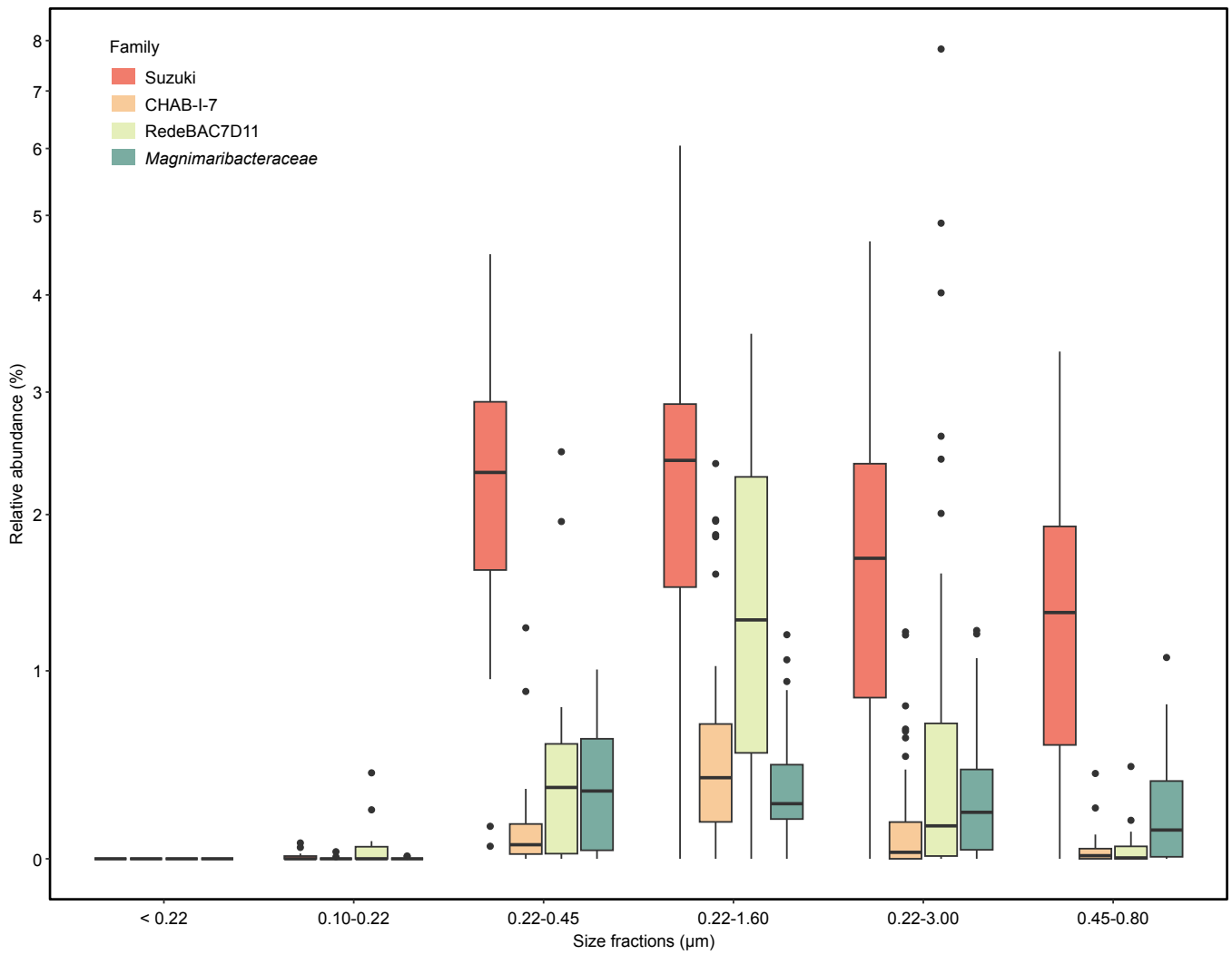

Supplement: ramfelt_supplementary_Figure_4_wrae227 [file ramfelt_supplementary_figure_4_wrae227.pdf]

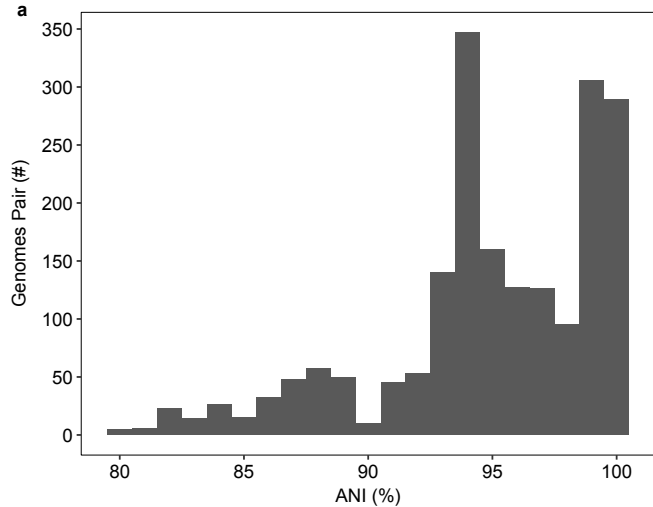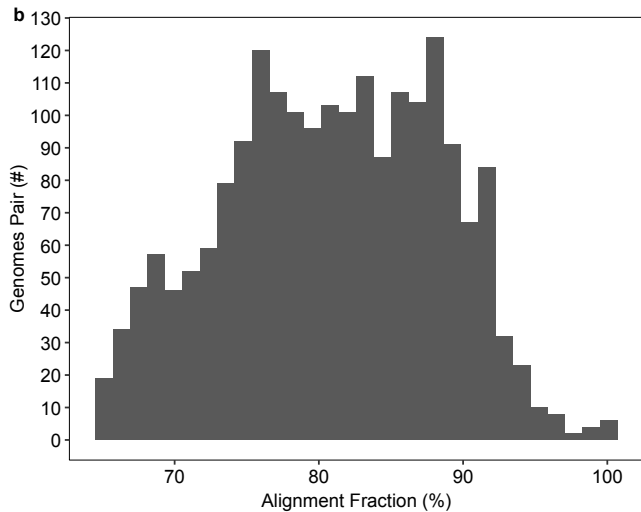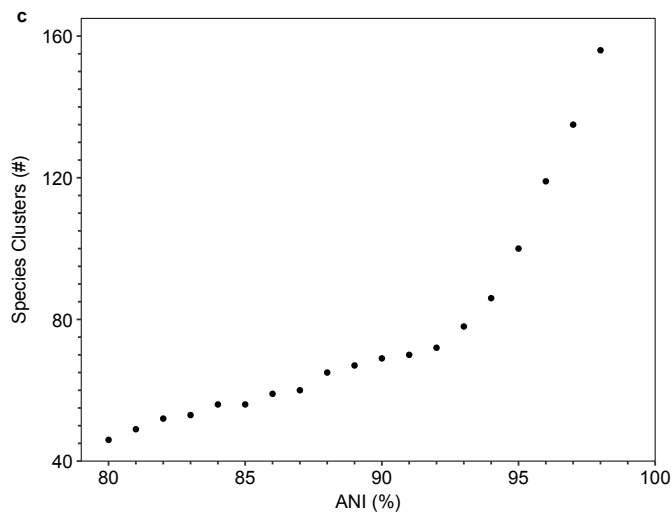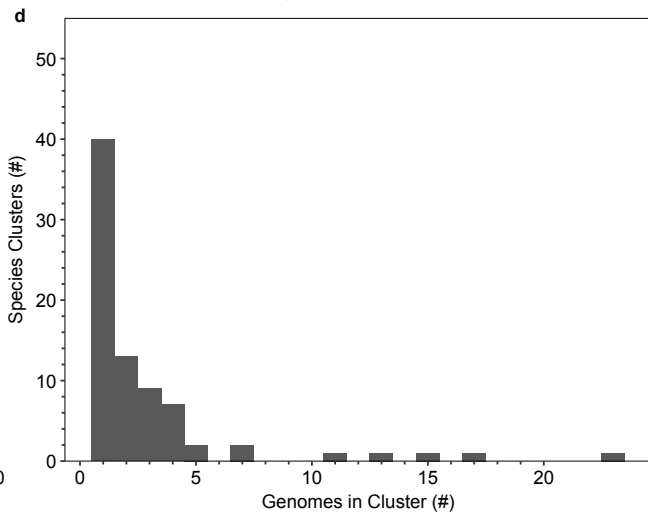

Supplement: ramfelt_supplementary_Figure_5_wrae227 [file ramfelt_supplementary_figure_5_wrae227.pdf]
