# Supplementary material for: Isolate-anchored comparisons reveal evolutionary and functional differentiation across SAR86 marine bacteria
Source: ISME J. 2024 Nov 9;18(1):wrae227. doi: 10.1093/ismejo/wrae227 (PMC11582366; doi:10.1093/ismejo/wrae227)
Supplement: ramfelt_supplementary_11042024 [file ramfelt_supplementary_11042024.docx]

# Supplementary Methods

## Electron microscopy

For preparation of specimens for transmission and scanning electron microscopy (TEM and SEM), 500 μL of cryopreserved HIMB1674 cells were defrosted at 20ºC and fixed with glutaraldehyde (final concentration, 2.5%). The sample was concentrated 10-fold using a pre-rinsed Amicon Ultra-15, 30 kDa MWCO (Millipore Sigma) at 14,000 x g for 10 min, recovered per manufacturer instructions, and diluted to 80 μL in sterile seawater.

For TEM, 40 μL of sample was deposited onto a glow-discharged, formvar-coated mesh grid using an air-driven ultracentrifuge (Airfuge CLS, Beckman) as previously described [1]. Briefly, the grid was secured to the distal interior surface of the Airfuge rotor chamber (EM-90, Beckman) and samples were centrifuged for 24 min at 20 PSI. After centrifugation, grids were stained with 0.02 μm-filtered 2% uranyl acetate. Grids were examined with a Hitachi HT7700 transmission electron microscope with 100 kV accelerating voltage. High resolution images were taken using an AMT BioSprint16M-Active Vu 16 megapixel cooled 4896 x 3920 pixel CCD camera.

For SEM, fixed HIMB1674 cells were loaded onto 13 mm diameter, 0.1 μm pore-sized polycarbonate filters, washed twice with 0.1 M sodium cacodylate buffer with 0.44 M sucrose, postfixed with 1% OsO4 in 0.1 M sodium cacodylate, and finally dehydrated through a graded ethanol series (30%, 50%, 70%, 85%, 95%, 100%). Filters were submerged in 1:2 EtOH/HMDS (hexamethyldisilazane) for 40 min, then left in 100% HMDS overnight until dry. Filters were mounted on aluminum stubs with double-stick conductive carbon tape and coated with gold/palladium in a Hummer 6.2 sputter coater. Specimens were viewed and digital images were acquired with a Hitachi S-4800 Field Emission Scanning Electron Microscope at an accelerating voltage of 5 kV. TEM and SEM micrographs were analyzed using ImageJ software [2]. Cell biovolume was calculated using the equation for prolate spheroid cells [3] : $V=\pi/6\cdot d^{2}\cdot w$

##

## PCR amplification and phylogenetic analysis of the full length SSU rRNA gene

Approximately 35 picograms of genomic DNA from HIMB1674 was used as template for PCR amplification (Bio Rad C1000 Touch; Bio Rad, Hercules, CA, USA) using the primers 27FB [4] and R1492 [5] that targeted the full length 16S rRNA gene. The 25 μL reaction volume contained 1 μL of genomic DNA template, 0.5 μL of each forward and reverse primer (final concentration of 0.2 μM), 10 μL of 5PRIME HotMasterMix (Quantabio, Beverly, MA, USA), and 13 μL of H2O. The reaction included an initial denaturing step of 2 min at 94°C, followed by 35 cycles of 30 s at 95°C, 1 min at 51°C, and 45 sec at 72°C, and a final extension of 12 min at 72°C. The resulting PCR product was cleaned (QIAquick PCR Purification Kit), Sanger sequenced in the forward and reverse directions, and assembled into a single contig.

The full length 16S rRNA gene from strain HIMB1674 was imported into the ARB software package [6] along with those extracted from the environmental genomes of the SAR86 expanded dataset where available. The genes were aligned using SINA v.1.2.11 [7] to a curated database of marine 16S rRNA gene sequences based on Silva v.95 [8], and phylogenetic analyses were performed using the RAxML maximum likelihood method with the GTR model of nucleotide substitution under the gamma and invariable- models of rate heterogeneity [9]. Bootstrap analysis (1,000 replicates) was performed using the rapid bootstrap analysis algorithm of RAxML. A set of *Betaproteobacteria* 16S rRNA gene sequences from cultured isolates were used as an outgroup.

##

## Evaluation of “complete composite genomes” reported by Roda-Garcia et al.

We used multiple approaches in an attempt to verify that the “complete composite genomes” (CCGs) reported by Roda-Garcia and colleagues are circularized and single contig as reported [10]. First, we attempted to repeat the assembly of CCGs from their respective SAGs using the program Flye v2.9 [11] with the arguments ‘-genome size 1.3m -subassemblies’ as reported by Roda-Garcia and colleagues. Of the nine assemblies, three were circularized and single contig, one was a single open contig, and six contained multiple contigs (Table S8). We also attempted to verify the validity of the nine original CCGs reported by [10] using a sequence mapping approach. First, we fragmented the original SAGs used to assemble each CCG into 10 kb fragments with 1 kb overlap, and 1 kb fragments with 100 bp overlap, using the tool cut_up_fasta.py from CONCOCT [12]. We then mapped the resulting 10 kb and 1 kb fragments to their respective CCG separately and in combination, and inspected the mapping manually. Numerous mis-assemblies were clearly evident across each of the nine CCGs, ranging from 6 in TMED112-A1 to 31 in D2474-A1-2 (Table S8). Close inspection revealed that, at least in this case of this study, the Flye assembler had joined sequences erroneously. The Flye Github documentation also revealed that the ‘-subassemblies’ option is not as thoroughly tested as the primary workflows and was deprecated in v2.9. Given the convincing evidence that the CCGs of [10] are chimeric artifacts, we were compelled to use the native SAGs generated by [13] in our analyses.

##

## Phylogenomic analyses

To create species clusters, the program fastANI v1.32 [14] was first used to calculate “average nucleotide identity” (ANI) between all quality-filtered *Magnimaribacterales* order genomes. A histogram of ANI values identified a peak in ANI at 93-94% (Fig. S5), and so an ANI value of 93% was used to group the *Magnimaribacterales* genomes into species clusters. In order to create species clusters, the genomes were sorted from maximum to minimum percent completeness. Then, starting with the most complete genome, a species cluster was created by identifying all other *Magnimaribacterales* order genomes with >93% ANI relative to this representative. After removing the clustered genomes from the rank completion list, the genome with the next highest completion value was used in the same manner to create a species cluster. Through this iterative process, all quality-controlled genomes were grouped into individual species clusters that each possessed a representative genome of the highest completion.

In order to create the Proteobacteria-wide phylogeny with the SAR86 species dataset, marker genes for the SAR86 species dataset were first identified using the “Genome Taxonomy Database Toolkit” (GTDB-Tk) v1.7.0 ‘identify’ program [15], which used Prodigal v2.6.3 [16] to call genes and HMMER v3.1b2 [17] to identify the 120 bacterial marker genes used by GTDB [18]. GTDB-Tk ‘align’ was then used to create a multiple sequence alignment of the SAR86 species dataset and GTDB-identified reference genomes for every species cluster within the GTDB phylum Proteobacteria using the 120 marker genes identified above. IQ-Tree v2.1.2 was used to perform a phylogenomic analysis using this alignment file with the LG4X+F model [19]. Using the large phylogeny as a guide, Treemmer v0.3 [20] was subsequently implemented to prune the tree to 1,000 genomes, with the conditions that at least two genomes from each family of Proteobacteria were retained (if available) and no representative genomes of the *Magnimaribacterales* order were removed. The genomes retained in the pruned genome alignment were used as input for GTDB-Tk ‘identify’, GTDB-Tk ‘align’ (with the ‘-–skip_gtdb_refs’ flag), and IQ-Tree (LG4X+F model). This phylogeny was then used as the guide tree to calculate site frequency profiles for another phylogenomic analysis that employed a site-specific frequency model (model Poisson+UDM0064NONE+G4). This analysis used the UDM 64 component model with no transformations (UDM0064NONE) from the Homology-Derived Structures of Proteins (HSSP) database [21], with IQ-Tree used to construct the phylogeny. This phylogeny was inspected to identify duplicate genomes, genomes labeled as “SAR86” but evolutionarily unaffiliated with the SAR86 lineage, and MAGs outside of the *Magnimaribacterales* order that possessed anomalously long terminal branches. After removal of these genomes, 849 remained (Table S3). The phylogeny produced using the UDM 64 site-specific frequency model was subsequently regenerated after removing the anomalous genomes, using 1,000 ultrafast bootstrap replicates.

To identify comparable taxonomic levels across sublineages of the *Magnimaribacterales*, the *Magnimaribacterales* species dataset was used. A relative evolutionary distance approach was then used to identify taxonomic levels within the SAR86 lineage. A bacterial domain-level phylogeny was created using the GTDB-Tk ‘de_novo_workflow’ [15] with the SAR86 expanded dataset and “p__Chloroflexota’’ as the outgroup. This workflow identified marker genes in the input genomes and aligned them using the same methods previously described. The workflow then inferred a tree using FastTree v2.1.10 (model WAG+GAMMA) [22], rooted the tree on the specified outgroup taxon (p__Chloroflexota), and decorated the internal nodes using the GTDB taxonomy. This phylogeny was then used as the input for the scale_tree program in PhyloRank (v0.1.11, <https://github.com/dparks1134/PhyloRank>) to convert branch lengths into relative evolutionary distance (RED). RED values of 0.82 and 0.9237 were used to identify family and genus-level lineages, respectively. These values were based on the distribution of internal nodes within the *Magnimaribacterales* order clade, their support values, and values used previously for other family and genus-level lineages [18].

##

## Read Recruitment

Metagenomic read recruitment was used to investigate the abundance of *Magnimaribacterales* order genomes across two publicly available open-ocean sampling endeavors: TARA Oceans [23] and GEOTRACES [24]. Within the TARA Oceans study, only metagenomes sequenced from the 0.22-3 μm size fraction were used. To prevent closely related genomes from competing for reads, only the SAR86 species dataset was used. Sequence reads were quality-filtered using ‘iu-filter-quality-minoche’ from the illumina-utils program v2.12 [25]. Bowtie2 v2.4.2 [26] was used to map reads from the metagenomes to the genomes. SAMtools v1.11 [27] was used to sort and index SAM files into BAM files. To obtain detection estimates, a contig database was created from the concatenated genomes and used to profile each of the BAM files using ‘anvi-profile’ from the anvi’o program suite v7.1 [28]. These profiles were then merged into a single profile database. Contigs in the merged profile were linked back to their associated genome using published methods [29,30]. The merged profile was then summarized using ‘anvi-summarize’ to obtain statistics on detection and abundance for the genomes in every metagenome.

The approach of [31] was used to estimate the abundance of *Magnimaribacterales* order genomes, calculated by taking the number of mapped reads divided by the total number of quality controlled reads found for each metagenome. These values were then divided by the size of each genome. This calculation was also performed for reads that did not map to any of the *Magnimaribacterales* order genomes. For this unidentified fraction, an average genome size of 1.6 Mbp was used, calculated from the relative abundance and genome sizes found by [13]. Relative abundances were estimated by summing together the normalized abundances of the *Magnimaribacterales* order genomes and the unidentified fraction for each metagenome. The sum was then divided from each normalized abundance estimate to generate the relative abundance of the genome or the unidentified fraction in the metagenome. To correct for genomes that had low detection values (i.e. breadth of coverage), the relative abundance of any genomes that had a detection value below 50% was defaulted to 0, indicating that the genome was not present in the associated metagenome. This detection threshold value was based on an analysis previously performed on *Prochlorococcus* populations [29]. To calculate the relative abundance of family and genus-level lineages within the *Magnimaribacterales* order, the relative abundance of each genome within each family and genus-level lineage was summed.

##

## Pangenomics

The anvi’o pangenome workflow v7.1 [28,29] was used with the SAR86 expanded genome dataset to identify patterns in gene content. A contig database was created for each *Magnimaribacterales* order genome and annotated with clusters of orthologous genes (COGs) [32] and Kyoto’s Encyclopedia of Genes and Genome [33] using ‘anvi-run-ncbi-cogs’ and ‘anvi-run-kegg-kofams’, respectively. The module ‘anvi-run-hmms’ was used to identify a collection of bacterial single copy core genes, which identified open reading frames using Prodigal [16] and identified them via HMMER [17]. Subsequently, a genome storage database was generated using the program ‘anvi-gen-genomes-storage’. The genome storage database was used as input for the pangenomic analysis using the command ‘anvi-pan-genome’ with the parameters “–minbit 0.5”, “–mcl-inflation 2”, and “–use-ncbi-blast”. This command used BLAST [34] to examine similarity between pairs of genes using the translated DNA sequence and the Markov Cluster algorithm [35] to form homologous gene clusters.

#

# Supplementary Results

## Both the TCA and methylcitrate cycles are important components of central carbon metabolism in SAR86 bacteria

All *Magnimaribacterales* families contain the components necessary for a functional TCA cycle (Table S5). Although genomes of the *Magnimaribacterales* lacked a gene for citrate synthase, they contained a 2-methylcitrate gene that can fulfill the same function as citrate synthase that can also support the 2-methylcitrate cycle. The Suzuki and CHAB-I-7 families both contained genes encoding the components of the 2-oxoglutarate dehydrogenase complex responsible for the conversion of 2-oxoglutarate to succinyl-CoA; however, these were largely absent in *Magnimaribacteraceae* and RedeBAC7D11 which instead possessed a 2-oxoacid oxidoreductase complex that likely fulfills this function (Fig. 3, Table S5) [36]. General features of *Magnimaribacterales* genomes also include both components of the glyoxylate shunt as well as a complete 2-methylcitrate cycle, except for the CHAB-I-7 family which lacked a gene encoding a methylisocitrate lyase needed to complete 2-methylcitrate cycle. This suggests that CHAB-I-7 may lack the capacity to convert methylisocitrate to pyruvate and succinate.

Although, most components of the Embden-Meyerhof-Parnas (EMP) glycolytic pathway were present across the *Magnimaribacterales*, a gene encoding glucose-6-phosphate isomerase was absent from many RedeBAC7D11 and *Magnimaribacteraceae* genomes (Fig. 3, Table S5). RedeBAC7D11 also lacked a pyruvate kinase, suggesting that this family may not have the capacity to convert phosphoenolpyruvate to pyruvate. The genes required for gluconeogenesis were present across all families of the *Magnimaribacterales* (Fig. 3, Table S5). Although most genomes from the Suzuki and CHAB-I-7 families contained genes encoding the components for a pyruvate dehydrogenase complex, RedeBAC7D11 and *Magnimaribacteraceae* lacked genes encoding the E1 and E2 components. Instead, these two families may utilize the same 2-oxoacid oxidoreductase complex used in the TCA cycle in order to convert pyruvate into acetyl-CoA (Fig. 3, Table S5).

##

## *Magnimaribacterales* genomes harbor genes to produce bacteriorhodopsin but not all-trans-retinal

Genes encoding proteorhodopsin were ubiquitous across the *Magnimaribacterales* order except for Ma-G1 and Ma-G2 of the *Magnimaribacteraceae* family (Fig. 3, Table S5). All genomes of the *Magnimaribacterales* appear to lack the ability to synthesize retinal *de novo*. Although predicted all-trans-8’-apo-beta-carotenal 15,15’-oxygenases found across the *Magnimaribacteraceae* and within some genomes of the RedeBAC7D11 family suggests at least a portion of the *Magnimaribacterales* order is capable of cleaving β-carotene to produce retinal, the presence of these functions in *Magnimaribacterales* order genomes that lack the gene for proteorhodopsin raises the possibility that it serves an alternative function.

## Historical perspective on the relationship between SAR86 and SAR156

The first description of SAR156 appeared in 1995 [37], wherein Mullins and colleagues unambiguously (and accurately) included SAR156 within the SAR86 clade. Unfortunately, subsequent studies either (i) excluded SAR156 from under the broader SAR86 umbrella and designated SAR156 as its own clade (e.g. [38,39]), (ii) excluded SAR156 completely (e.g. [40]), or (iii) properly included SAR156 as part of the SAR86 clade (e.g. [41]). Some bad luck was also involved: the original SAR86 16S rRNA gene was chimera, the original SAR156 16S rRNA gene was a partial sequence, and one prominent publication that referred to SAR86 and SAR156 [40] provided incorrect citations regarding SAR86 subclade structure.

Despite this backstory, genome phylogenies and a more “filled out” phylogenomic tree unambiguously show SAR156 to be one of four major subgroups of SAR86, and is reflected in both Silva (16S rRNA gene) and GTDB (genome) databases, albeit with confusing nomenclature.

# References

1. Brum JR, Steward GF. Morphological characterization of viruses in the stratified water column of alkaline, hypersaline Mono Lake. *Microb Ecol* 2010;**60**:636–43. https://doi.org/10.1007/s00248-010-9688-4

2. Schneider CA, Rasband WS, Eliceiri KW. NIH Image to ImageJ: 25 years of image analysis. *Nat Meth* 2012;**9**:671–5 https://doi.org/10.1038%2Fnmeth.2089

3. Hillebrand H, Dürselen CD, Kirschtel D, Pollingher U, Zohary T. Biovolume calculation for pelagic and benthic microalgae. *J Phycol* 1999;**35**:403–24. https://doi.org/10.1046/j.1529-8817.1999.3520403.x

4. Vergin KL, Urbach E, Stein JL, DeLong EF, Lanoil BD, Giovannoni SJ. Screening of a fosmid library of marine environmental genomic DNA fragments reveals four clones related to members of the order *Planctomycetales*. *App Environ Microbiol* 1998;**64**:3075–8. https://doi.org/10.1128/AEM.64.8.3075-3078.1998

5. Lane D. 16S/23S rRNA sequencing. *Nucleic Acid Techniques in Bacterial Systematics*. New York, NY: John Wiley & Sons, 1991, p 115–175.

6. Ludwig W, Strunk O, Westram R, Richter L, Meier H, Yadhukumar A et al. ARB: a software environment for sequence data. *Nucleic Acids Res* 2004;**32**:1363–71. https://doi.org/10.1093/nar/gkh293

7. Pruesse E, Peplies J, Glöckner FO. SINA: accurate high-throughput multiple sequence alignment of ribosomal RNA genes. *Bioinformatics* 2012;**28**:1823–9. https://doi.org/10.1093/bioinformatics/bts252

8. Quast C, Pruesse E, Yilmaz P, Gerken J, Schweer T, Yarza P et al. The SILVA ribosomal RNA gene database project: improved data processing and web-based tools. *Nucleic Acids Res* 2012;**41**:D590–6. https://doi.org/10.1093/nar/gks1219

9. Stamatakis A. RAxML-VI-HPC: maximum likelihood-based phylogenetic analyses with thousands of taxa and mixed models. *Bioinformatics* 2006;**22**:2688–90. https://doi.org/10.1093/bioinformatics/btl446

10. Roda‐Garcia JJ, Haro‐Moreno JM, Rodriguez‐Valera F, Almagro‐Moreno S, López‐Pérez M. Single-amplified genomes reveal most streamlined free-living marine bacteria. *Environ Microbiol* 2023;**25**:1136–54. https://doi.org/10.1111/1462-2920.16348

11. Kolmogorov M, Yuan J, Lin Y, Pevzner PA. Assembly of long, error-prone reads using repeat graphs. *Nat Biotechnol* 2019;**37**:540–6. https://doi.org/10.1038/s41587-019-0072-8

12. Alneberg J, Bjarnason BS, De Bruijn I, Schirmer M, Quick J, Ijaz UZ et al. Binning metagenomic contigs by coverage and composition. *Nat Meth* 2014;**11**:1144–6. https://doi.org/10.1038/nmeth.3103

13. Pachiadaki MG, Brown JM, Brown J, Bezuidt O, Berube PM, Biller SJ et al. Charting the complexity of the marine microbiome through single-cell genomics. *Cell* 2019;**179**:1623–1635.e11. https://doi.org/10.1016/j.cell.2019.11.017

14. Jain C, Rodriguez-R LM, Phillippy AM, Konstantinidis KT, Aluru S. High throughput ANI analysis of 90K prokaryotic genomes reveals clear species boundaries. *Nat Commun* 2018;**9**:5114. https://doi.org/10.1038/s41467-018-07641-9

15. Chaumeil PA, Mussig AJ, Hugenholtz P, Parks DH. GTDB-Tk: a toolkit to classify genomes with the Genome Taxonomy Database. *Bioinformatics* 2020;**36**:1925–7. https://doi.org/10.1093/bioinformatics/btz848

16. Hyatt D, Chen GL, LoCascio PF, Land ML, Larimer FW, Hauser LJ. Prodigal: prokaryotic gene recognition and translation initiation site identification. *BMC Bioinformatics* 2010;**11**:119. https://doi.org/10.1186/1471-2105-11-119

17. Eddy SR. Accelerated profile HMM searches. *PLOS Comput Biol* 2011;**7**:1–16. https://doi.org/10.1371/journal.pcbi.1002195

18. Parks DH, Chuvochina M, Waite DW, Rinke C, Skarshewski A, Chaumeil PA et al. A standardized bacterial taxonomy based on genome phylogeny substantially revises the tree of life. *Nat Biotechnol* 2018;**36**:996–1004. https://doi.org/10.1038/nbt.4229

19. Minh BQ, Schmidt HA, Chernomor O, Schrempf D, Woodhams MD, Von Haeseler A et al. IQ-TREE 2: new models and efficient methods for phylogenetic inference in the genomic era. *Mol Biol Evol* 2020;**37**:1530–4. https://doi.org/10.1093/molbev/msaa015

20. Menardo F, Loiseau C, Brites D, Coscolla M, Gygli SM, Rutaihwa LK et al. Treemmer: a tool to reduce large phylogenetic datasets with minimal loss of diversity. *BMC Bioinformatics* 2018;**19**:164. https://doi.org/10.1186/s12859-018-2164-8

21. Schrempf D, Lartillot N, Szöllősi G. Scalable empirical mixture models that account for across-site compositional heterogeneity. *Mol Biol Evol* 2020;**37**:3616–31. https://doi.org/10.1093/molbev/msaa145

22. Price MN, Dehal PS, Arkin AP. FastTree 2 – approximately maximum-likelihood trees for large alignments. *PLOS ONE* 2010;**5**:1–10. https://doi.org/10.1371/journal.pone.0009490

23. Sunagawa S, Coelho LP, Chaffron S, Kultima JR, Labadie K, Salazar G et al. Structure and function of the global ocean microbiome. *Science* 2015;**348**:1261359. https://doi.org/10.1126/science.1261359

24. Biller SJ, Berube PM, Dooley K, Williams M, Satinsky BM, Hackl T et al. Marine microbial metagenomes sampled across space and time. *Sci Data* 2018;**5**:180176. https://doi.org/10.1038/sdata.2018.176

25. Eren AM, Vineis JH, Morrison HG, Sogin ML. A filtering method to generate high quality short reads using illumina paired-end technology. *PLOS ONE* 2013;**8**:1–6. https://doi.org/10.1371/journal.pone.0066643

26. Langmead B, Salzberg SL. Fast gapped-read alignment with Bowtie 2. *Nat Methods* 2012;**9**:357–9. https://doi.org/10.1038/nmeth.1923

27. Danecek P, Bonfield JK, Liddle J, Marshall J, Ohan V, Pollard MO et al. Twelve years of SAMtools and BCFtools. *GigaScience* 2021;**10**, https://doi.org/10.1093/gigascience/giab008

28. Eren AM, Kiefl E, Shaiber A, Veseli I, Miller SE, Schechter MS et al. Community-led, integrated, reproducible multi-omics with anvi’o. *Nat Microbiol* 2021;**6**:3–6. https://doi.org/10.1038/s41564-020-00834-3

29. Delmont TO, Eren AM. Linking pangenomes and metagenomes: the Prochlorococcus metapangenome. *PeerJ* 2018;**6**:e4320. https://doi.org/10.7717/peerj.4320

30. Delmont TO, Kiefl E, Kilinc O, Esen OC, Uysal I, Rappé MS et al. Single-amino acid variants reveal evolutionary processes that shape the biogeography of a global SAR11 subclade. *eLife* 2019;**8**:e46497. https://doi.org/10.7554/eLife.46497

31. Shaiber A, Willis AD, Delmont TO, Roux S, Chen LX, Schmid AC et al. Functional and genetic markers of niche partitioning among enigmatic members of the human oral microbiome. *Genome Biol* 2020;**21**:292. https://doi.org/10.1186/s13059-020-02195-w

32. Galperin MY, Makarova KS, Wolf YI, Koonin EV. Expanded microbial genome coverage and improved protein family annotation in the COG database. *Nucleic Acids Res* 2015;**43**:D261–9. https://doi.org/10.1093/nar/gku1223

33. Ogata H, Goto S, Sato K, Fujibuchi W, Bono H, Kanehisa M. KEGG: Kyoto encyclopedia of genes and genomes. *Nucleic Acids Res* 1999;**27**:29–34. https://doi.org/10.1093/nar/27.1.29

34. Altschul SF, Gish W, Miller W, Myers EW, Lipman DJ. Basic local alignment search tool. *J Mol Biol* 1990;**215**:403–10. https://doi.org/10.1016/S0022-2836(05)80360-2

35. Enright AJ, Van Dongen S, Ouzounis CA. An efficient algorithm for large-scale detection of protein families. *Nucleic Acids Res* 2002;**30**:1575–84. https://doi.org/10.1093/nar/30.7.1575

36. Dörner E, Boll M. Properties of 2-oxoglutarate:ferredoxin oxidoreductase from *Thauera aromatica* and its role in enzymatic reduction of the aromatic ring. *J Bacteriol* 2002;**184**:3975–83. https://doi.org/10.1128/JB.184.14.3975-3983.2002

37. Mullins TD, Britschgi TB, Krest RL, Giovannoni SJ. Genetic comparisons reveal the same unknown bacterial lineages in Atlantic and Pacific bacterioplankton communities. *Limnol Oceanogr* 1995;**40**:148–58. https://doi.org/10.4319/lo.1995.40.1.0148

38. DeLong EF, Preston CM, Mincer T, Rich V, Hallam SJ, Frigaard NU et al. Community genomics among stratified microbial assemblages in the ocean’s interior. *Science* 2006;**311**:496–503. https://doi.org/10.1126/science.1120250

39. Suzuki MT, Preston CM, Béjà O, De La Torre JR, Steward GF, DeLong EF. Phylogenetic screening of ribosomal RNA gene-containing clones in bacterial artificial chromosome (BAC) libraries from different depths in Monterey Bay. *Microb Ecol* 2004;**48**:473–88. https://doi.org/10.1007/s00248-004-0213-5

40. Treusch AH, Vergin KL, Finlay LA, Donatz MG, Burton RM, Carlson CA et al. Seasonality and vertical structure of microbial communities in an ocean gyre. *ISME J* 2009;**3**:1148–63. https://doi.org/10.1038/ismej.2009.60

41. Kan J, Evans SE, Chen F, Suzuki MT. Novel estuarine bacterioplankton in rRNA operon libraries from the Chesapeake Bay. *Aquat Microb Ecol* 2008;**51**:55–66. https://doi.org/10.3354/ame01177

# Supplementary Figures

Figure S1. Relative Evolutionary Distance (RED)-scaled *Magnimaribacterales* phylogeny indicating family and genus designations. This phylum-wide analysis was performed using the Genome Taxonomy Database Toolkit (GTDB-Tk). Only the subtree for the *Magnimaribacterales* order is shown. The green vertical line indicates the family-level RED value of 0.82, while the purple line indicates the genus-level RED value of 0.9237.

Figure S2. Ribosomal RNA-based phylogeny of the family *Magnimaribacteraceae*. Filled circles indicate major nodes with bootstrap support >90%, whereas colors indicate depth of sampling in the water column. The scale bar corresponds to 0.02 substitutions per nucleotide position. A selection of *Betaproteobacteria* were used as an outgroup.

Figure S3. Distribution of GC content across genera of the four families of *Magnimaribacterales*. Color of box plots indicate the family that they belong to and the x axis indicates their genus.

Figure S4. Relative abundance of metagenomic reads across the four *Magnimaribacterales* families within different size fractionated TARA Oceans metagenomes (n=204). Color of box plots indicate the family that each boxplot represents. The x axis indicates the different size fractionations that were present in the TARA oceans metagenomes used.

Figure S5. Characteristics of pair-wise comparisons between quality filtered genomes of the SAR86 order *Magnimaribacterales* (n=224). a, Histogram of ANI values (%) among all quality filtered SAR86 genomes, revealing a peak at 93-94%. b, Histogram of the alignment fraction (%) between all quality filtered SAR86 genomes. c, Number of species clusters created for different ANI values. d, Histogram of the distribution of genomes within each species cluster using ANI value of 93%.

# Supplementary Tables

Table S1. Detection of the 280 gene markers included within the CheckM *Gammaproteobacteria* set across *M. mokuoloeensis* str. HIMB1674 and 731 putative environmental SAR86 genomes. Markers included in the second iteration of the CheckM workflow are indicated in the HIMB1674_marker_set column.

Table S2. Characteristics of 732 putative environmental SAR86 genomes. The SAR86_expanded column indicates genomes that were used in the SAR86 expanded dataset, whereas the SAR86_species_rep column indicates genomes used in the SAR86 species dataset. NA indicates data elements that were not included in publicly available metadata.

Table S3. Genomes used to create the Proteobacteria-wide phylogenomic analysis shown in Fig. 1b. Taxonomy classifications are from GTDB release 202. N/A indicates no available taxonomic classification.

Table S4. Metagenomes used to quantify the distribution of SAR86 genomes via read recruitment. Oceanic regions correspond to those used in Figure 4. The columns read_pairs_raw and read_pairs_passed indicate the number of raw reads in each sample and the number of reads that passed quality control respectively.

Table S5. Distribution of genes responsible for core metabolic pathways and functions across genomes of the SAR86 order *Magnimaribacterales*. Columns ending with “Mean” indicate average copy number and those ending with “Proportion” indicate proportion of genomes with at least one copy. A column is present for each genus, as well as for individual *M. mokuoloeensis* HIMB1674 and Pelagibacter str. HIMB083 genomes.

Table S6. Data underlying the estimated relative abundance of each family within the SAR86 order *Magnimaribacterales* that corresponds to Figure 4c.

Table S7. Protologue for *Magnimaribacter mokuoloeensis* str. HIMB1674.

Table S8. Summary of assembly errors in CCGs (complete composite genomes) reported by [22].
